# Land use change reshaped soil carbon dynamics in Northern China

**DOI:** 10.1371/journal.pone.0359439

**Published:** 2026-10-09

**Authors:** Yuanyuan Tang, Yuchun Yang, Guohong Du, Yanbing Qi

**Affiliations:** 1 College of Resources and Environment, Xichang University, Xichang, Sichuan, China; 2 College of Natural Resources and Environment, Northwest A&F University, Yangling, Shaanxi, China; Shandong University, CHINA

## Abstract

Northern China has experienced some of the most intensive land use changes globally, yet the consequent impacts on soil carbon dynamics and regional source-sink patterns, particularly the divergent responses of soil organic carbon (SOC) and soil inorganic carbon (SIC), remain poorly quantified. This study examined how land use change reshapes SOC and SIC pools and modulates their sensitivity to environmental drivers. Using nationwide soil survey data from the 1980s and 2010s, we applied random forest models and partial least squares structural equation modeling to disentangle the responces SOC and SIC pools for land use and environmental change. Results revealed that Northern China functioned as a net soil carbon source over the 30-year interval, losing approximately 0.56 Gt of SOC and 0.56 Gt of SIC. Land use type emerged as the dominant control on carbon source-sink patterns. Grassland transitioned into strong carbon sources driven primarily by SIC losses of 1.51 Gt, whereas unutilized land exhibited estimated net carbon gains with apparent SIC accumulation of 2.06 Gt. Beyond altering carbon stocks, land use change reversed regional source-sink patterns and markedly heightened the sensitivity of soil carbon dynamics to environmental changes with explanatory strength increment of 0.11 for SOC and 0.09 for SIC, respectively. Specifically, SOC responded primarily to biotic drivers such as vegetation productivity, while SIC exhibited tighter coupling with increasing atmospheric CO_2_ fluctuations. The main contribution of this study is revealing how SOC and SIC respond differently to environmental drivers following land use change. Practically, these results indicate that regional carbon management strategies must fully consider land use change effects and explicitly include soil inorganic carbon in carbon neutrality accounting frameworks.

## Introduction

Over the past 60 years, nearly one-third of the global land cover has undergone changes in land use type [1]. Changes in the northern hemisphere are primarily attributed to afforestation and farmland abandonment, while changes in the southern hemisphere are dominated by deforestation and farmland expansion [2]. China has also similarly experienced dramatic land use change over the past four decades. Extensive grassland cover northern regions, where significant pasture expansion has occurred, but overgrazing has also led to widespread grassland degradation [3,4].

The regulatory role of land use change on soil carbon pool is now widely recognized [3,5]. As the largest active carbon pool in terrestrial ecosystems, soil carbon pools (SCP) primarily consist of organic and inorganic carbon components [6]. Soil organic carbon (SOC) has long attracted the bulk of research interest, largely because it responds quickly to land management and directly exchanges CO_2_ with the atmosphere [7,8]. Researchers have extensively documented how SOC storage shifts when land use changes, whether through deforestation, cultivation, or afforestation. Meta-analyses broadly agree that restoring vegetation tends to boost SOC sequestration. However, in the absence of additional anthropogenic carbon inputs, degrading grasslands or converting land to agriculture often leads to significant carbon losses [9]..

However, soil inorganic carbon (SIC), which comprises primarily carbonate minerals, has historically been treated as a relatively stable, geogenic pool with limited biogeochemical relevance on management timescales [10]. This perception began to shift only in recent years, as accumulating evidence showed that SIC is in fact dynamic and responsive to environmental forcing [11,12]. For instance, a global meta-analysis of land use change effects on SIC revealed that conversions from grassland to farmland can increase SIC by up to 30.6%, whereas grassland restoration may reduce it by 14.4%, demonstrating that SIC responses are directionally opposite to those of SOC in many contexts [10]. Parallel to land use effects, climate-change factors have also been shown to influence SIC dynamics: regional drought events and elevated atmospheric CO_2_ concentrations can promote inorganic carbon accumulation via accelerated carbonate precipitation and the CO_2_ fertilization effect, respectively [13–15].

Collectively, these findings suggest that SOC and SIC do not respond independently. Rather, they exist in a state of dynamic equilibrium that regulates the net direction of soil carbon sources and sinks [16]. Despite the well-documented individual responses of SOC and SIC to land use and climate change, the soil carbon source/sink effects arising from the dynamic equilibrium of these two components have been consistently overlooked and underquantified. For instance, large-scale vegetation restoration projects have been implemented in Northern China to enhance carbon sequestration, but if grazing intensity is not properly regulated, soil carbon loss driven by grassland degradation could offset the sequestration benefits achieved through restoration in some areas [16]. Therefore, to thoroughly investigate how soil carbon sources and sinks respond to abrupt land use change within the broader context of environmental change, it is essential to systematically elucidate the dynamics of the SCP by quantifying the coupled responses of organic and inorganic carbon pools during land use conversions.

Achieving this quantification is complicated by the fact that land use change never operates in isolation. It interacts continuously with climate, biota, and topography, the key soil-forming factors that govern SCP dynamics through long-term inputs and weathering processes [17,18]. The response of SCP to these interacting drivers involves complex biogeochemical mechanisms that cannot be captured by examining land use alone. Consequently, fully quantifying the impact of multiple drivers on SCP dynamics necessitates embedding the coupled SOC–SIC framework within a more precise and systematic assessment system.

In early investigations of soil carbon dynamics, stepwise multiple regression and Pearson correlation analysis were widely used to identify key controlling factors. Researchers relied on these approaches to show that climate and topographic variables often dominate SOC spatial variation, and to quantify the statistical links between land use and carbon stocks [19,20]. More recently, the Geodetector model has been adopted to explore factor interactions, using its interaction detector to reveal two-factor enhancement or nonlinear synergistic effects [21]. Still, these methods are fundamentally geared toward parsing relative contributions; they do not adequately capture the path-dependent relationships and synergistic mechanisms among multiple drivers [22].

To bridge this gap, structural equation modeling (SEM) has gradually been brought into ecological and soil science research. SEM was originally developed and widely applied in the social sciences [5,23], but it has since been adapted to test causal pathways in vegetation-soil-climate systems. However, conventional SEM relies on maximum likelihood estimation and imposes stringent demands on sample size, requirements that are frequently unmet in regional-scale soil surveys [24]. In this study, partial least squares SEM (PLS-SEM) offers a more practical alternative, especially when sample sizes are limited and multiple latent variables are present [25]. It is important to recognize that PLS-SEM has inferential limitations compared with ML-SEM, such as potential biases in path estimations, the lack of global goodness-of-fit indices, and sensitivity to indicator selection [26,27]. Even so, when the research objective emphasizes prediction and theory development rather than strict theory confirmation, PLS-SEM proves useful for dissecting SCP dynamics driven by multifactorial interactions [27].

This study aims to investigate the response of SCP dynamics to intense land use type change under the influence of global and environmental change. It seeks to clarify the role of land use change in soil carbon processes and quantify the contributions of environmental and land use change to SCP dynamics. Specifically, based on soil sample data from the 1980s and 2010s, this research employs a random forest (RF) feature selection framework to spatially predict SOC and SIC. Building upon this foundation, the study integrates SOC and SIC to estimate regional SCP in Northern China. Subsequently, a PLS-SEM is employed to couple soil carbon dynamics with climate changes (encompassing biotic, climatic, and nitrogen deposition) under different land use changes. This approach aims to reveal the dynamic evolution of SCP and its sources/sinks under the dual drivers of intense human activities and complex environmental changes, thereby achieving the research objectives. Based on the above research background and purpose, this study proposes the following core assumptions:

(1) From the 1980s to the 2010s, the soil carbon pool in Northern China acted as a net carbon source, but its intensity exhibited significant spatial heterogeneity. Ecological projects such as the Grain-for-Green Program are expected to promote SOC accumulation, whereas farmland expansion in some areas may induce SIC losses; the net effect of these opposing processes remains uncertain. Considering the dramatic land use changes across Northern China, which may enhance the dynamics and vulnerability of soil carbon pools, this 30-year temporal window captures a critical transitional phase during which land use change is expected to induce measurable reallocation and redistribution of carbon across pools and soil depths, reflecting such enhanced dynamics. Therefore, we hypothesize that the SCP in Northern China will function as a net carbon source over this transient, policy-relevant period rather than reflecting a stabilized pedogenic equilibrium.
(2) Different types of land use change are expected to exert contrasting effects on SOC and SIC. The Grain-for-Green Program will significantly increase SOC, but is expected to have no significant effect on SIC. Conversely, cropland expansion (especially dryland farming) will significantly reduce SIC, whereas SOC may show no significant change or only a slight increase, suggesting that SIC may serve as a more sensitive early indicator of land use change impacts in these arid soils at decadal timescales.
(3) Global climate change regulates soil carbon dynamics through multiple pathways, and the intensity of its effects depends on the type of land use change. Specifically, the CO_2_ fertilization effect is expected to enhance vegetation productivity, which in turn will positively affect SOC through increased litter input, but is expected to have no significant effect on SIC. Regional drought may negatively affect SOC accumulation through reduced vegetation productivity, but its effects on SIC accumulation are expected to vary with drought intensity: moderate drying may promote carbonate precipitation via reduced leaching, but severe drought may disrupt ion mobility and precipitation kinetics, leading to either neutral or negative effects on SIC depending on soil water potential thresholds.

## Materials and methods

### Study area

Northern China comprises 17 provinces (municipalities and autonomous regions, Fig 1a). Only certain areas of some provinces such as Shaanxi, Henan, Anhui, and Jiangsu were included in the study area based on the boundary defined by the Qinling Mountains and the Huai River. The region covers a total area of 5.58 million km², accounting for 58% of the land area of mainland China’s land area. The annual average temperatures range from −22℃ to 16℃, and the precipitation amount varies between 150 and 1400 mm. Due to global climate change, the climatic conditions in Northern China transformed significantly from the 1980s to the 2010s, where the average annual temperature increased by 1.92°C and the annual precipitation decreased by 8.16 mm [16]. The overall topography varies from higher elevations in the west and gradually decreasing toward the east. The agricultural climate zones in Northern China are characterized by a highly complex spatial pattern. The western part of the study area mostly comprises Arid and Semi-Arid Regions such as Xinjiang and Gansu, as well as the Qinghai-Tibet Plateau. The central part encompasses the Loess Plateau and highly intensive agricultural zones represented by the Guanzhong Plain and the Huang-Huai-Hai Plain. The eastern part is predominantly covers the Middle-Lower Yangtze Plain and the Northeast Plain.

**Fig 1:**
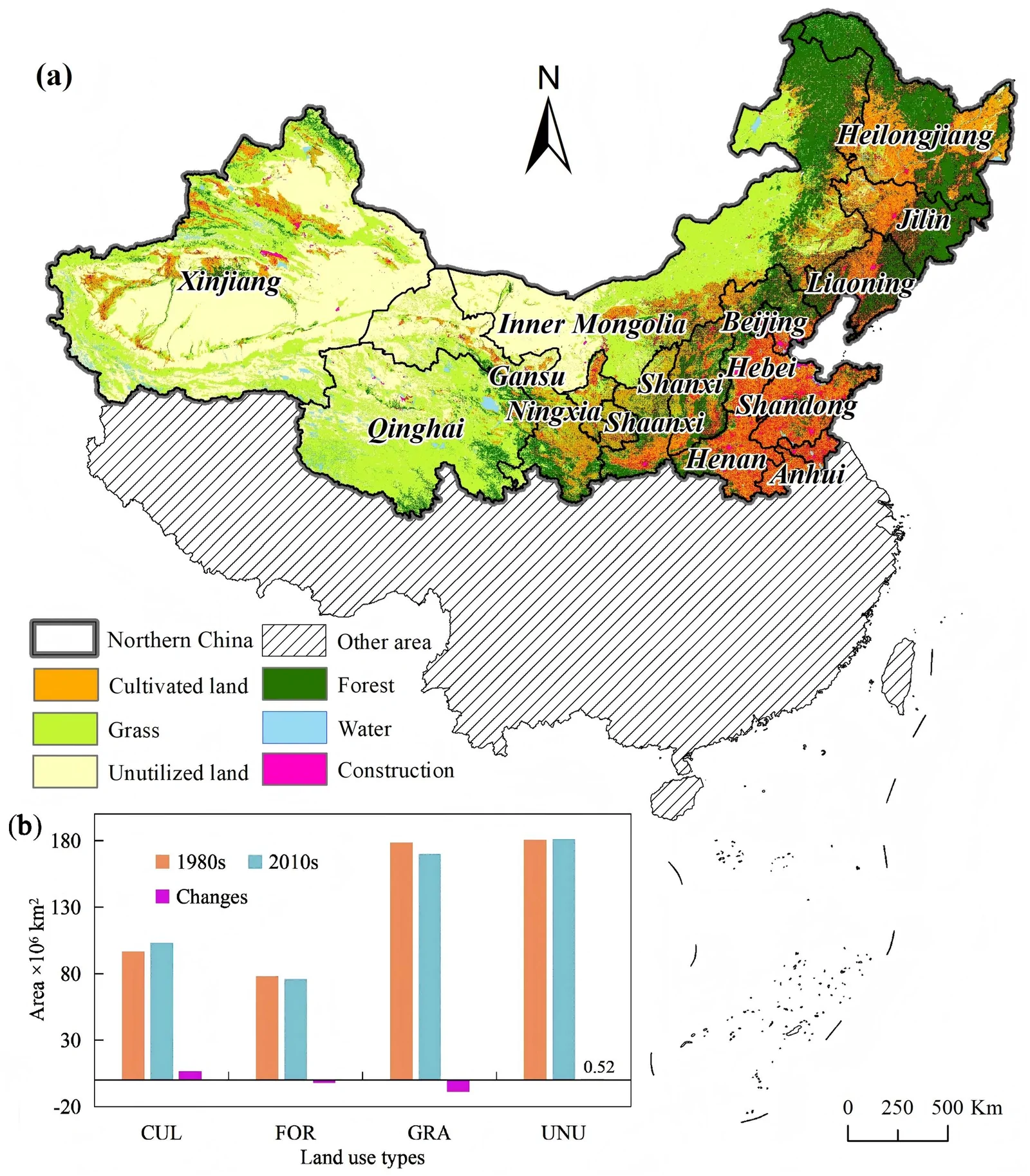
Study area. “CUL”, “FOR”, “GRA”, and “UNU” represent cultivated land, forest, grassland, and unutilized land.

Northern China is one of the regions that have experienced the most intense land use change globally [28], where approximately 22.29% of the land has undergone significant shifts in land use type. Compared with Southern China, the land use patterns in Northern China have undergone more pronounced changes, particularly in ecologically fragile agricultural and pastoral areas [9]. Extensive areas of cultivated land (CUL), forest (FOR), grassland (GRA), and unutilized land (UNU) are distributed in Northern China. Given that water bodies and urban construction land operate under distinct anthropogenic and hydrological mechanisms and account for only 3.25% of the total area change, this study focuses on natural and semi-natural land use transitions (CUL, FOR, GRA, and UNU). From 1985 to 2015, CUL and GRA underwent a significant inverse evolution (Fig 1b).

### Data sources

#### Sources of SOC and SIC data.

The soil carbon data from the 1980s used in this study were derived from the second national soil survey conducted in every province of China. The survey results included data regarding the specific locations of typical soil profiles, soil layer depth, elevation, soil color, bulk density, soil mechanical composition, organic matter content, and calcium carbonate content. The soil carbon data for the 2010s used in this study were derived from a nationwide soil survey (Fig 2). Similarly, the results obtained in this study encompassed data regarding the latitude and longitude of representative profiles, bulk density, soil texture, organic matter content, and calcium carbonate content. Data utilized regarding the latitude, longitude, bulk density, gravel (> 2 mm) content, organic matter content, and calcium carbonate content from representative profiles within a series of findings were obtained from surveys conducted in the 1980s and 2010s to separately determine SOC and SIC stocks [29].

**Fig 2:**
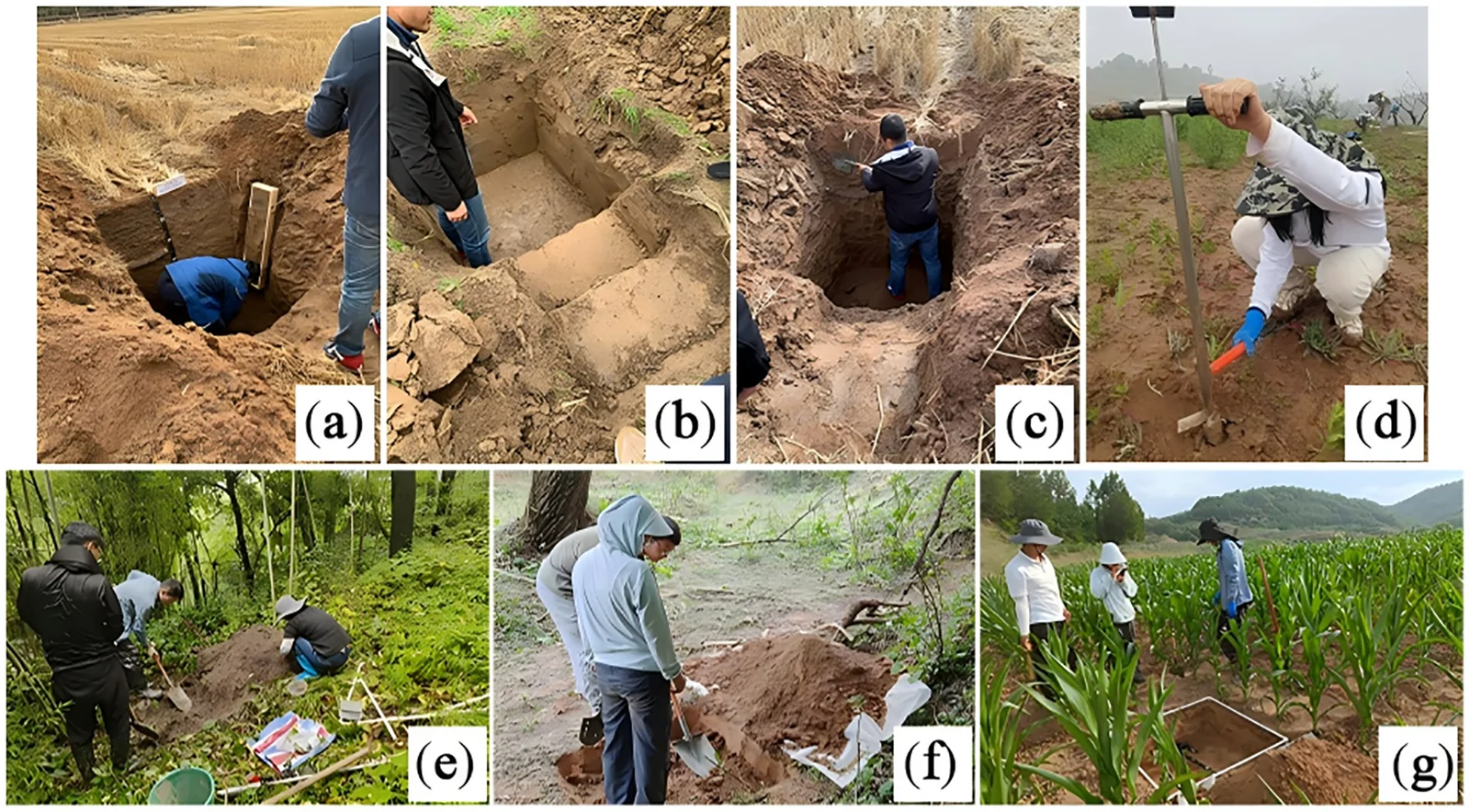
Field soil sampling. (a) Soil sampling profile of CUL. (b) Sampling profile. (c) Sampling profile. (d) Soil sampling by drilling. (e) Soil sampling of FOR. (f) Soil sampling of GRA. (g) Soil sampling of CUL. The 1980s soil profile data were extracted from the published provincial Soil Species Records, which were compiled by local agricultural stations during the Second National Soil Survey of China. For the 2010s campaign, soil sampling was conducted by the authors on publicly accessible land, or on farmland and grassland with prior permission from the local agricultural stations and the individual land users. None of the sampling sites were located within protected areas, and the fieldwork involved only small-volume, non-destructive soil sampling with no impact on endangered or protected species. Therefore, no additional fieldwork permits were required.

As shown in Fig 3, the soil sampling point density for the 1980s and 2010s covered all land use types, as well as encompassing all agricultural climate zones. Table 1 shows a details of the numbers of sampling points across all land use types, demonstrating that the number of sampling points on CUL far exceeded that on other land use types. In addition, among the soil sampling points from the 1980s used in this study, 3290 points had valid SOC data, and 1970 points had valid SIC data, and the numbers were 2043 and 1239, respectively, for the corresponding soil sampling points in the 2010s.

**Table 1:** Number of sampling points in different land use types.

Land use types ^a^	Age	
	Number	
	1980s	2010s
Cultivated land	1756	1060
Forest	383	217
Grass land	627	448
Water	80	38
Construction land	286	120
Unutilized land	158	160
Sum	3290	2043

^a^In this system, “Water” refers to land covered by permanent surface water, including rivers and canals, lakes, reservoirs and ponds, tidal flats, and glaciers/permanent snow; “Construction land” refers to artificially built and intensively used land, including urban built-up areas, rural settlements, and industrial, mining, and transportation infrastructure land.

**Fig 3:**
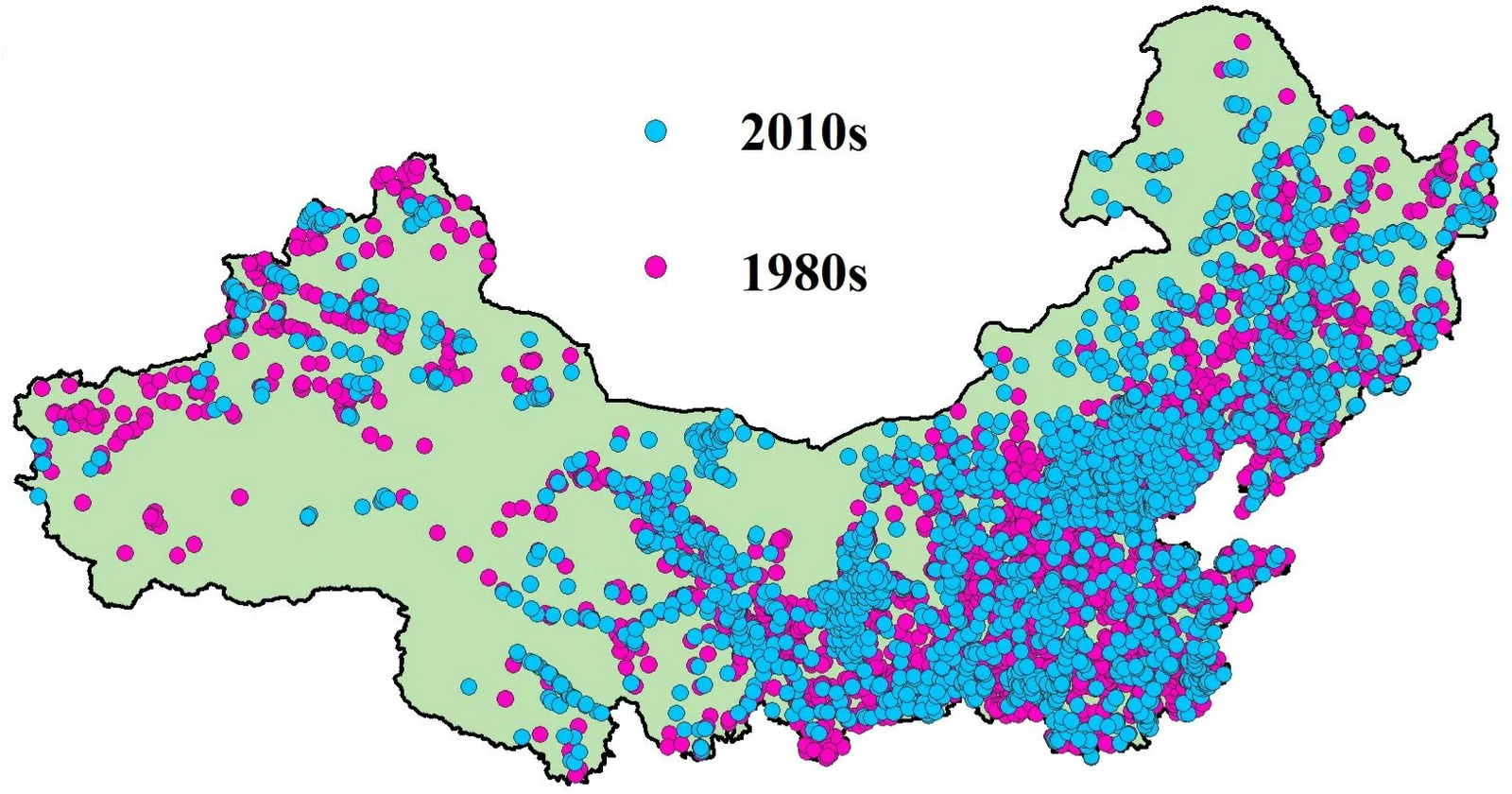
Spatial distribution of soil sampling points in the 1980s and 2010s. The data sources of the sampling sites are shown in Table 2.

**Table 2:** Framework of covariate data used in RF model.

Covariate data	Sources
Digital elevation model, DEM	Resource and Environmental Science and Data Platform (https://www.resdc.cn/)
Slope, SLO	
Aspect, ASP	
Accumulated surface temperature, GST ^a^	
Total annual precipitation, PRE ^a^	
Average annual temperature, TEM ^a^	
Average annual air pressure, AP ^a^	
Relative humidity, RHU ^a^	
Average annual sunshine duration, SSD ^a^	
Annual accumulated temperature, GDD ^b^	
Wind speed,WIN ^a^	
Actual annual evaporation, EVP ^a^	
Net primary production, NPP ^a^	Google Earth Engine, GEE (https://developers.google.cn/earth-engine/)
Normalized difference vegetation index, NDVI ^b^	
Fractional vegetation cover, FVC ^b^	
Climatic water scarcity, CWS	
Annual potential evapotranspiration, PET ^a^	
Actual evapotranspiration, AET ^a^	
Soil dryness index, DI ^a^	
Runoff, RO ^a^	
Soil water storage, SWS ^a^	
Soil bulk density, BD ^c^	Data for the 1980s were derived from the second national soil survey and data for the 2010s from the National Earth System Science Data Center (http://soil.geodata.cn/)
Cation exchange capacity, CEC ^c^	
pH ^c^	
NHx-N deposition, NHx ^b^	[30]
NOy-N deposition, NOy ^b^	
Volume fraction of coarse debris, VFCD ^c^	ISRIC (https://www.isric.org/explore/soilgrids)
Silt (< 0.002 mm) content, SILT ^c^	
Sandy (> 0.05 mm) content, SAND ^c^	
Clay (≥ 0.002 mm and ≤ 0.05 mm) content, CLAY ^c^	
Gravel (> 2 mm) content, GC ^c^	
Soil anoxic respiration, SAR ^b^	ORNL (https://daac.ornl.gov/SOILS/guides/SRDB_V5.html)
Soil respiration, Rs ^b^	
Soil saturation hydraulic conductivity, KSAT ^c^	HiHydroSoil v2.0 (https://www.futurewater.eu/projects/hihydrosoil/)

^a^These covariates were derived from the data of 1985 and 2015 to represent the 1980s and 2010s, respectively, due to 5-year interval constraints in climate data availability.

^b^These covariates were are multi-year means (1982/1983/1984–1986 for the 1980s with the starting year for the 1980s varying from 1982 to 1984 depending on data availability, and 2012–2016 for the 2010s).

^c^These data were originally organized and labeled by the data providers as the 1980s and 2010s.

#### Sources of covariate data for RF modeling.

A RF feature selection framework was rmployed to spatially predict SOC and SIC, incorporating 34 covariate factors (Table 2). The predictive performance of a RF model can be enhanced by increasing the tree strength and reducing the number of correlations between trees [30]. Therefore, it was necessary to screen the most suitable covariates for use in RF prediction and screening results are shown in Fig 4.

**Fig 4:**
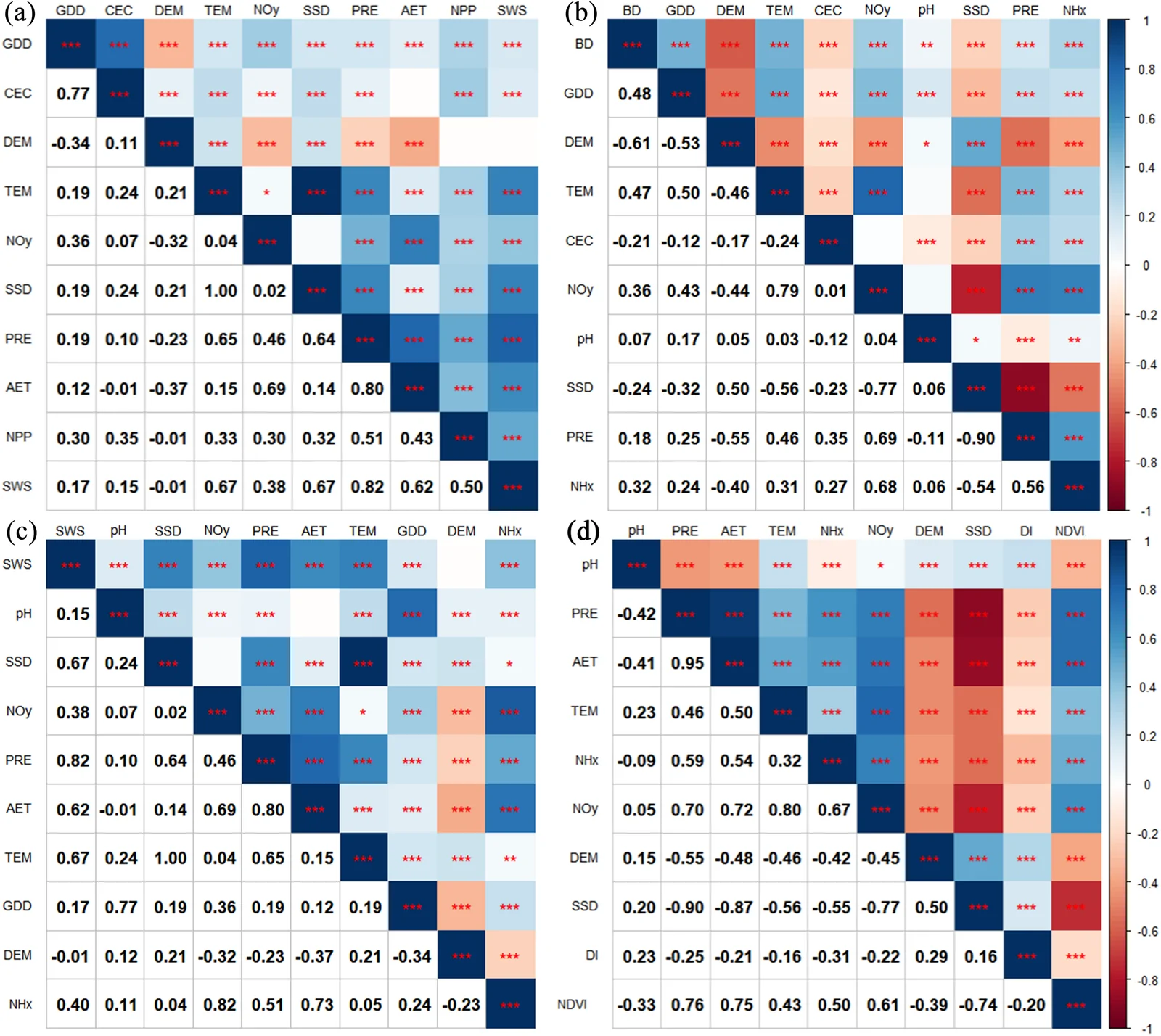
Correlation heat map of key factors. (a) 1980s SOC, (b) 2010s SOC, (c) 1980s SIC, and (d) 2010s SIC.

#### Sources of observed variables for PLS-SEM.

Based on a review of previous research and theoretical frameworks, key drivers of large-scale regional SCP dynamics were integrated into the following observed variables when constructing the PLS path model (Table 3). Biological latent variables were represented by the leaf area index and net primary productivity of vegetation. Nitrogen deposition latent variables were based on atmospheric and anthropogenic inputs of ammonium and nitrate nitrogen, as well as the soil pH, which is highly correlated with SIC and nitrogen inputs. The Nitrogen construct comprises NHx, NOy, and pH. While NHx and NOy represent direct nitrogen inputs, pH is included as a proximal regulator of nitrogen bioavailability and transformation processes, consistent with the soil nitrogen cycle framework. The Climate construct is operationalized as an integrated climate change factor comprising atmospheric CO_2_ (radiative forcing proxy) and regional hydrothermal indicators (TEM, PRE, and PDSI). These indicators reflect complementary facets of the same underlying climate change process at different spatial scales, consistent with coupled climate-carbon cycle frameworks. In addition, a series of landscape indices were selected, including comprehensive land use dynamics, average fractal dimension, maximum patch index, aggregation, Shannon diversity index, patch density, and landscape separation, were selected to comprehensively characterize latent variables reflecting land use and landscape pattern changes. This systematic approach to the construction of an analytical framework reflected multi-factor interactions.

**Table 3:** Observed variables for PLS-SEM.

Latent data	Observed data	Data Types	Sources
Biology	Leaf area index, LAI ^b^	Products	GEE (https://developers.google.cn/earth-engine/)
	Gross primary production, GPP, g m^−2^ (8 day)^−1 b^		
Nitrogen	NHx-N deposition, NHx, Tg ^b^		[31]
	NOy-N deposition, NOy, Tg ^b^		
	pH ^c^	Derived data	As shown in Table 2
Climate	Annual average temperature, TEM, ℃ ^a^		Climate data were dericed from the Ordinary Kriging (OK) interpolation results for meteorological stations from the Geographic Remote Sensing Ecological Network Platform (https://gisrs.cn/)
	Annual precipitation, PRE, mm ^a^		
	Annual accumulated temperature, GDD, ℃ ^b^		
	Palmer drought severity index, PDSI ^a^	Products	GEE (https://developers.google.cn/earth-engine/)
	CO_2_ concentration, % ^b^		EDGAR (https://edgar.jrc.ec.europa.eu/dataset_ghg2025)
Land use ^d^	Comprehensive land use dynamic degree, LC, %	Derived data	The original land use data for 1985 and 2015 were derived from the Resource and Environmental Science and Data Platform. Each index was calculated as explained in section 2.3.3.
	Fractal dimension, FRAC		
	Largest patch index, LPI, %		
	Landscape division index, Division		
	Shannon diversity index, SHDI		
	Aggregation index, AI, %		
	Patch density, PD, per hectare		

^a^These covariates were derived from the data of 1985 and 2015 to represent the 1980s and 2010s, respectively, due to 5-year interval constraints in climate data availability.

^b^These covariates were are multi-year means (1982/1983/1984–1986 for the 1980s with the starting year for the 1980s varying from 1982 to 1984 depending on data availability, and 2012–2016 for the 2010s).

^c^This data were originally organized and labeled by the data providers as the 1980s and 2010s.

^d^All land use metrics were calculated with 1985 as the base period and 2015 as the current period.

### Methods

#### Method for calculating soil carbon stocks.

To control for potential systematic biases arising from methodological differences, we ensured that the data for both the 1980s and 2010s were based on the typical soil profiles. Consistent technical protocols were followed throughout the entire process of sample collection, preservation, and physicochemical property determination process. The data from both decades covered the surface organic matter layer (typically comprising the litter layer, humus layer, and O-layer). However, the aims of this study were to quantify and analyze carbon storage and its dynamics in mineral soils, so all data for the surface O-layer data were excluded during initial data screening. Only SCP data from mineral soil horizons (e.g., Aa-layer, Ap-layer, P-layer, Br-layer) were retained. Therefore, the SOC content was determined by using the *Walkley-Black* method and converted into the soil organic matter content using the *Van Bemmelen* conversion factor of 1.724 in the 1980s and 2010s [32,33]. The calcium carbonate equivalent content was quantified using a volumetric method. The SIC content was derived by applying stoichiometric conversion factors, especially the mass ratio of carbon in calcium carbonate (12/100 = 0.12) [34].

The field sampling depths for typical profile sites generally exceeded 100 cm. To extend soil profiles with a sampling depth of < 100 cm to a uniform depth of 100 cm, we employed an equal-area spline function [35]. For profiles with measured depths of < 100 cm, the spline curve was linearly extrapolated from the deepest measured soil layer down to 100 cm. The soil carbon contents were obtained for depths of 0–20 cm, 20–40 cm, 40–60 cm, 60–80 cm, and 80–100 cm. A cross-validation approach was adopted to evaluate the accuracy of spline interpolation and extrapolation, where complete profiles with measured depths ≥ 100 cm were artificially truncated to shallower depths. Missing data below the truncation depth were predicted using the spline function combined with linear extrapolation, and the predictions were then compared against the originally measured values (Fig 5). The concordance correlation coefficient (CCC, Formula 1) and Bias (Formula 2) were also selected as evaluation indices to measure the precision and accuracy of the model. Furthermore, Monte Carlo simulations were additionally employed to perform uncertainty analysis for depth extrapolation, aiming to quantify the potential impact range of profile extrapolation errors on the regional estimates of SCP, SOC, and SIC stocks.

**Fig 5:**
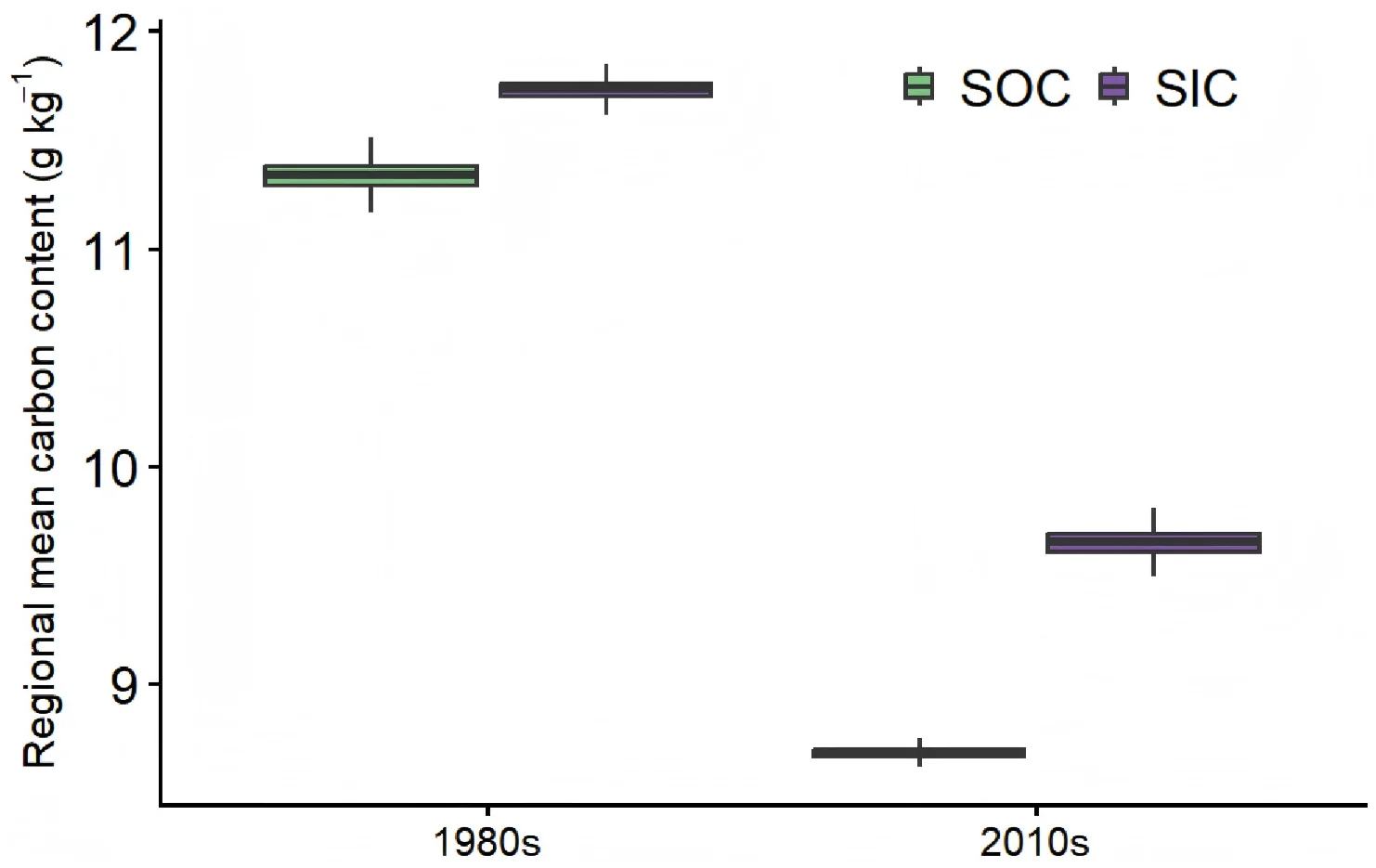
Monte Carlo uncertainty of the equal-area spline extrapolation (N = 2000). N is the number of simulations, SD is the standard deviation, CV is the coefficient of variation, P2.5 and P97.5 is the percentiles, and Relative 95% CI width is the relative confidence interval width.


$$\begin{array}{c} CCC=\frac{2\cdot Cov\left(x,y\right)}{\sigma_{x}^{2}+\sigma_{y}^{2}+{\left(\overset{―}{x}-\overset{―}{y}\right)}^{2}} \end{array}$$ (1)

$$\begin{array}{c} Bias=\frac{1}{\text{n}}\sum_{i=1}^{n}\left(y_{i}-x_{i}\right) \end{array}$$ (2)

In the above formulae, *x*_*i*_ is the mean of the observed values, and *y*_*i*_ is the predicted value. *¯x* is the mean of the observed values, and *¯y* is the mean of the predicted values. *σ*_*x*_^*2*^ is the variance of the observed value, and *σ*_*y*_^*2*^ is the variance of the predicted values. *Cov(x, y)* is the correlation coefficient between the observed value and the predicted value, and *n* is the number of samples. A positive *Bias* (> 0) indicates a systematic tendency toward overestimation, wherein the predicted values are larger than their observed counterparts. A negative *Bias* (< 0) indicates a systematic underestimation. There is no systematic bias when Bias ≈ 0.

Using an average thickness weighting method (Formula 3) [29,36], the SOC and SIC contents were obtained at a soil depth of 1 m. Subsequently, density and stock formulae (Formulae 4–5) were used to calculate the organic and inorganic carbon stocks [37], respectively. The sum of these two components constitutes the SCP. The changes in the soil carbon stocks from the 1980s to the 2010s were then analyzed (Formula 6). An increase in soil carbon stocks (*Q*_*c*_ > 0, Formula 7) indicates carbon sequestration in soil as a carbon sink, whereas a decrease (*Q*_*c*_ < 0) denotes a carbon source [34].


$$\begin{array}{c} SC_{0-100}=\sum SC_{i}\times \frac{H_{i}}{100} \end{array}$$ (3)

$$\begin{array}{c} SOCD\left(SICD\right)=BD\times SC_{0-100}\times D\times (1-GC)\times 0.01 \end{array}$$ (4)

$$\begin{array}{c} SOC\left(SIC\right)=A\times SOCD\left(SICD\right) \end{array}$$ (5)

$$\begin{array}{c} SCP=SOC+SIC \end{array}$$ (6)

$$\begin{array}{c} Q_{c}=SCP_{2010s}-SCP_{1980s} \end{array}$$ (7)

In the above formulae, *SC*_*0–100*_ denotes the organic or inorganic carbon content (g kg^−1^) of soil at a depth of 1 m, *SC*_*i*_ represents the carbon content (g kg^−1^) of the corresponding soil layer *i*, and *H*_*i*_ indicates the thickness (cm) of soil layer *i*. *SOCD* and *SICD* denote the organic and inorganic carbon density (kg m^−2^), respectively. *BD* represents the bulk density (g cm^−3^), *D* denotes the soil depth (0–100 cm), and *GC* is the gravel content (> 2 mm, %). *SOC* and *SIC* represent organic and inorganic carbon stocks (kg, 1 Gt = 10^12^ kg), respectively, where *A* denotes landscape or pixel area (m²). *SCP* denotes the soil carbon pool, where *SCP*_*1980s*_ and *SCP*_*2010s*_ represent the soil carbon pools in the 1980s and 2010s, and *Q*_*c*_ is used to reflect soil carbon sources or sinks.

#### RF feature selection and prediction framework.

RF has been shown to outperform conventional prediction models when fitting samples of organic carbon content [38,39], this study extends its successful application paradigm including the same core covariate set and optimized model parameters to SIC prediction. Variable screening was performed using the built-in importance assessment function of the *randomForest* package in R 4.5.2 [30]. Predictor variables were ranked by their mean decrease in gini scores, which quantify the total reduction in node impurity attributable to each variable across all trees. Variables in the top 10 were retained as core covariates for the final predictive model. To ensure stability, importance scores were averaged across all trees in the optimized forest. Model hyperparameters (ntree = 500, mtry = 3) were optimized using the grid search method provided by the *caret* package [39], with 10-fold cross-validation repeated five times to ensure generalization capability. To mitigate potential overfitting and multicollinearity, a systematic, iterative correlation screening was conducted among the RF-importance-screened variables (Fig 2). Variable pairs with Pearson’s |r| > 0.9 were identified; one variable from each pair was randomly removed, and the correlation matrix and importance ranking were recalculated for the remaining variables. This procedure was repeated iteratively until all remaining variable pairs exhibited |r| ≤ 0.9, yielding a final set of independent, non-redundant predictors. A fixed random seed was used to ensure full reproducibility of the variable ranking.

The accuracy of the RF model was measured using three commonly employed regression model evaluation metrics: root mean square error (RMSE, Formulae 8–9), mean absolute error (MAE, Formula 10), and R-squared (R², Formula 11). In Formulae 8–11, *y*_*i*_ denotes the true value, *y*_*RF*_ represents the predicted value, *ȳ* is the average of *y*_*i*_, and *n* indicates the sample size.


$$\begin{array}{c} MSE=\frac{\text{1}}{n}{\sum_{i=1}^{n}\left(y_{i}-y_{RF}\right)}^{\text{2}} \end{array}$$ (8)

$$\begin{array}{c} RMSE=\sqrt{MSE} \end{array}$$ (9)

$$\begin{array}{c} MAE=\frac{\text{1}}{n}\sum_{i=1}^{n}\left|y_{i}-y_{RF}\right| \end{array}$$ (10)

$$\begin{array}{c} R^{\text{2}}=1-\frac{\sum_{i=1}^{n}{\left(y_{i}-y_{RF}\right)}^{\text{2}}}{\sum_{i=1}^{n}{\left(y_{i}-\overset{―}{y}\right)}^{\text{2}}} \end{array}$$ (11)

In addition, spatial validation was required considering the scale of the study and that strong spatial structure may have existed in the data. Before performing spatial cross-validation, the sampling points were divided into several spatial blocks, where one of the blocks was used as the verification set, and the remaining blocks were used as the training set. In the process of spatial block partition process, the optimal number of blocks (k value) was determined by using the contour coefficient method combined with k-Means clustering, and then the *cluster* package in R was then used to perform the specific block partitioning [40]. In this study, Buffered Leave-One-Out based on the buffer radius was used for achieve spatial cross-validation to ensure that the training set and the validation set were spatially independent of each other. In particular, by using the *blockCV* package in R 4.5.2, the variogram of candidate covariates was calculated to quantify the range of their spatial autocorrelation, and the appropriate buffer distance is selected accordingly [41]. By taking the range of the variogram as the buffer radius, the training samples fwithin the buffer can be eliminated in order to evaluate the generalization ability of the RF model more fairly and effectively [42].

#### Quantitative land use change index.

To comprehensively assess the spatial patterns, process characteristics, and potential ecological effects of land use change, the following indicators were employed to construct a multidimensional, systematic analytical framework [13,43]. The composite land use dynamics index (0 ≤ LC < 100%) reflects the extent of land use change per unit area. The average fractal dimension index (1 ≤ FRAC ≤ 2) links the geometric shape of land use type patches to ecological processes. The largest patch index (0 ≤ LPI < 100%) measures the dominant land use type across different periods. The landscape division index (0 ≤ Division < 1) is a core metric for assessing the fragmentation or division of land use types. The Shannon diversity index (SHDI) fundamentally quantifies the compositional heterogeneity of land use types within a unit area. The aggregation index (0 ≤ AI < 100%) and patch density (0 ≤ PD < 100%) are two indicators for characterizing the spatial aggregation and spatial distribution density of land use types from different dimensions. A landscape unit of 100 × 100 pixels (3 × 3 km^2^) was used as the area unit. The specific indicator formulae used for calculating these indices are as follows:


$$\begin{array}{c} LC=\frac{\sum_{i=1}^{n}\Delta LU_{i-j}}{2\sum_{i=1}^{n}LU_{i}}\times \frac{\text{1}}{T}\times 100\% \end{array}$$ (12)

$$\begin{array}{c} FRAC=\frac{2ln\left(P\right)}{ln\left(A\right)} \end{array}$$ (13)

$$\begin{array}{c} LPI=\frac{Max\left(a_{ij}\right)}{A}\times 100\% \end{array}$$ (14)

$$\begin{array}{c} Division=1-{\sum_{i=1}^{n}\sum_{j=1}^{m}\left(\frac{a_{ij}}{A}\right)}^{\text{2}} \end{array}$$ (15)

$$\begin{array}{c} SHDI=-\sum_{i=1}^{m}\left(P_{i}\cdot lnP_{i}\right) \end{array}$$ (16)

$$\begin{array}{c} AI=\left(\frac{g_{ii}}{Maxg_{ii}}\right)\times 100\% \end{array}$$ (17)

$$\begin{array}{c} PD=\frac{N}{A}\times 1{\text{0}}^{\text{4}}\times 100 \end{array}$$ (18)

In the above formulae, *LC* denotes the comprehensive land use dynamics index, *FRAC* represents the average fractal dimension index, *LPI* represents the largest patch index, *Division* is the landscape division index, *SHDI* is the Shannon diversity index, *AI* is the aggregation index, and *PD* is the patch density, *△LU*_*i-j*_ represents the total area converted from land use type *i* to all other *non-i* land use types. *LU*_*i*_ represents the area of land use type *i* in 1985, with *T* set to 30, *P* is the patch perimeter, *A* is the patch area, and *N* is the total number of patches in the landscape. *a*_*ij*_ represents the area of patch *ij*, *A* represents the total landscape area, *P*_*i*_ is the proportion of patch type *i* relative to the total landscape area, and *m* is the number of patch types. *g*_*ii*_ is the number of adjacent pixels of the same type *i*, while *Max g*_*ii*_ denotes the maximum possible number of adjacent pixels for patch type *i*, and *100%* indicates the metric expressed as a percentage, whereas *100* denotes the number of patches per 100 ha.

#### Partial least squares structural equation modeling.

This study employed the *plspm* and *seminr* packages in R 4.5.2 to construct a partial least squares structural equation modeling (PLS-SEM) framework [44,45], thereby quantifying the hypothesized structural relationships among land use and environmental factors and the dynamics of organic and inorganic carbon pools. All constructs were specified as reflective. The measurement model comprised five exogenous constructs including Climate (PRE, PDSI, GDD, CO_2_, TEM), Nitrogen (NHx, NOy, pH), Land use (FRAC, SHDI, Division, PD, LPI, LC, AI), and Biology (LAI, GPP), and two endogenous single-indicator constructs (SOC and SIC dynamics under different land use changes). The structural model specifies the directed paths from the exogenous constructs to the endogenous constructs (Fig 6). Standardized path coefficients (*β*_*ji*_) were calculated following Formula 19 [27,46]:

**Fig 6:**
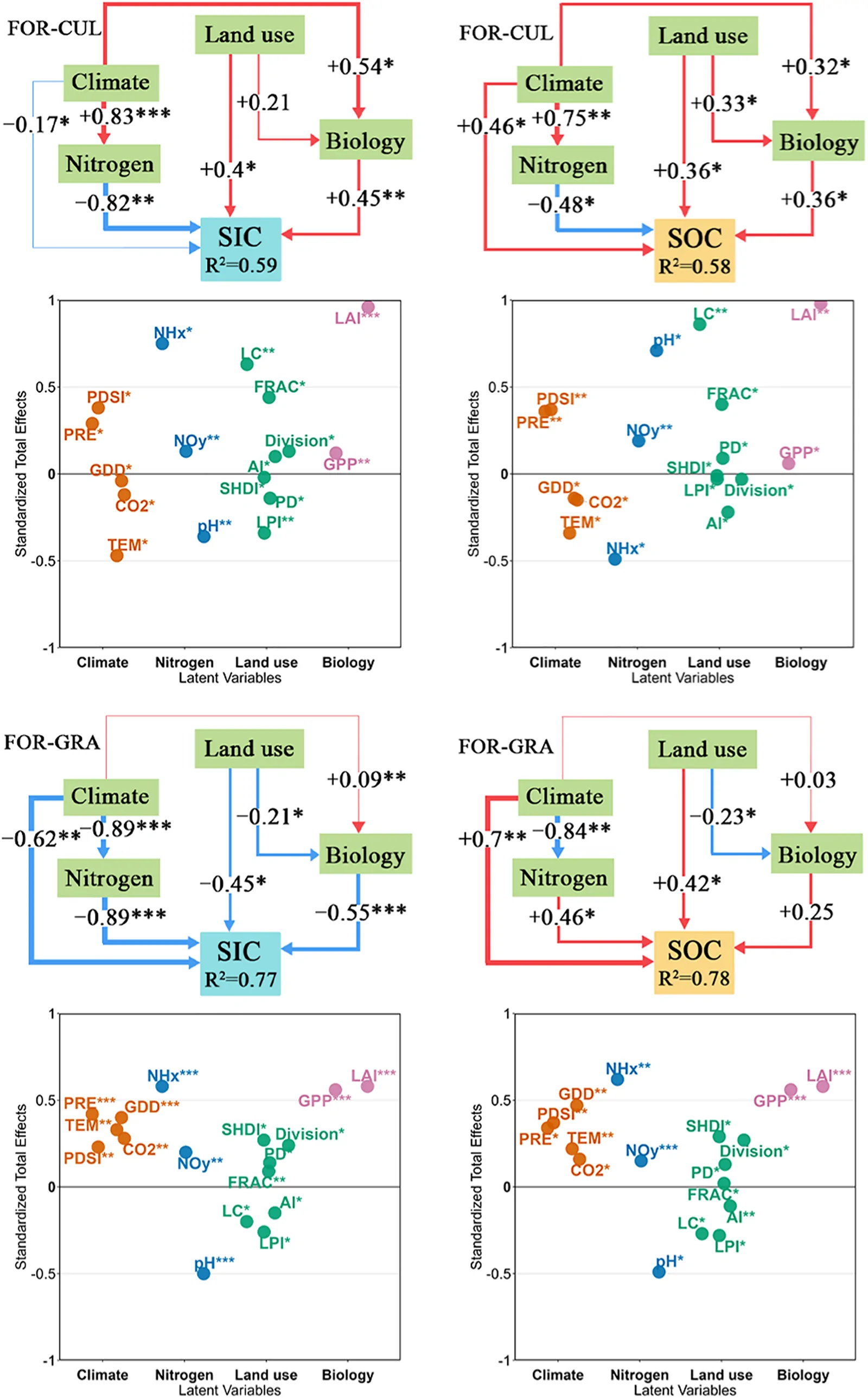
PLS-SEM for soil organic and inorganic carbon under two land conversion scenarios.


$$\begin{array}{c} \beta ji=\frac{Cov\left(\xi_{i},\eta_{j}\right)}{Var\left(\xi_{i}\right)}\times \frac{\sigma_{\xi i}}{\sigma_{\eta j}} \end{array}$$ (19)

In the above formula, *β*_*ji*_ represents the path coefficient between the latent variables *ξ*_*i*_ and latent variables *η*_*j*_. *σ* represents the standard deviation of latent variable scores. The statistical significance of all path coefficients was verified through 5000 bootstrap samples, and a 95% confidence interval excluding zero was considered statistically significant (α = 0.05). Model evaluation included Cronbach’s α (α), composite reliability (CR), average variance extracted (AVE), heterotrait-monotrait ratio (HTMT), and standardized root mean square residual (SRMR). The above indicators were computed using the *seminr* package in R 4.5.2 [45].

The variance inflation factor (VIF, Formula 20) was applied to assess multicollinearity within the formative measurement model and structural model [47]. When specifically applied to multicollinearity issues in PLS-SEM, VIF evaluates potential threats to model estimation stability arising from multicollinearity among indicators within the formative measurement model and among predictor variables pointing to the same endogenous variable within the structural model. R² (prediction effect value, R² in PLS-SEM explains the variance in latent variables to validate theoretical structures, whereas R² in the RF model explains variance in variables and is directly used for prediction, Formula 21) and Q² (prediction correlation, Formula 22) were employed to evaluate the model’s predictive power [48,49]. The formulae employed are defined as follows:


$$\begin{array}{c} VIF=\frac{\text{1}}{1-R_{j}^{\text{2}}} \end{array}$$ (20)

$$\begin{array}{c} R_{\eta j}^{\text{2}}=1-\frac{SSE_{\eta j}}{SST_{\eta j}} \end{array}$$ (21)

$$\begin{array}{c} Qcom,\eta j\text{2}=1-\frac{SSE_{com,\eta j}}{SSO_{com,\eta j}} \end{array}$$ (22)

In the above formulae, *R*^*2*^_*j*_ is the coefficient of determination obtained by regressing each observed variable under a latent variable against all other observed variables within that structure, *η*_*j*_ represents the endogenous latent variable, *SSE*_*ηj*_ is the sum of squares of deviations between the estimated value and its predicted value in the structural model, *SST*_*ηj*_ is the total variance of scores for the endogenous latent variable *η*_*j*_, *SSE*_*com,ηo*_ represents the sum of squares of deviations between actual values and model predictions for all manifest variables under latent variable *η*_*o*_, and *SSO*_*com,ηo*_ is the sum of squares of deviations between the actual values and mean values for all observed variables under latent variable *η*_*o*_. In general, *VIF* < 5 indicates no severe multicollinearity issues in the model, the value of *Q*^*2*^ > 0 shows that the model has predictive value, and *R²* closer to 1 denotes greater explanatory power, where *R²* > 0.1 indicates acceptable explanatory capacity [27,47].

## Results

### Spatio-temporal change in SCPs, carbon sources and sinks

From the 1980s to the 2010s, the soil carbon dynamics in Northern China played a significant role as a carbon source, with a net loss of 1.12 Gt form the carbon pools. In particular, both SOC and SIC stocks each lost approximately 0.56 Gt of carbon losses that SOC decreased from 48.24 ± 3.46 Gt in the 1980s to 47.68 ± 1.59 Gt in the 2010s, where the SIC stocks decreased from 43.43 ± 1.32 Gt to 42.87 ± 2.77 Gt.. As shown in Fig 7b, the severe imbalance between carbon sources and sinks was the primary factor related to the decline in regional soil carbon stocks. In particular, soil carbon sources exceeded sinks by 1.12 Gt, which precisely matched the total loss of carbon stocks.

**Fig 7:**
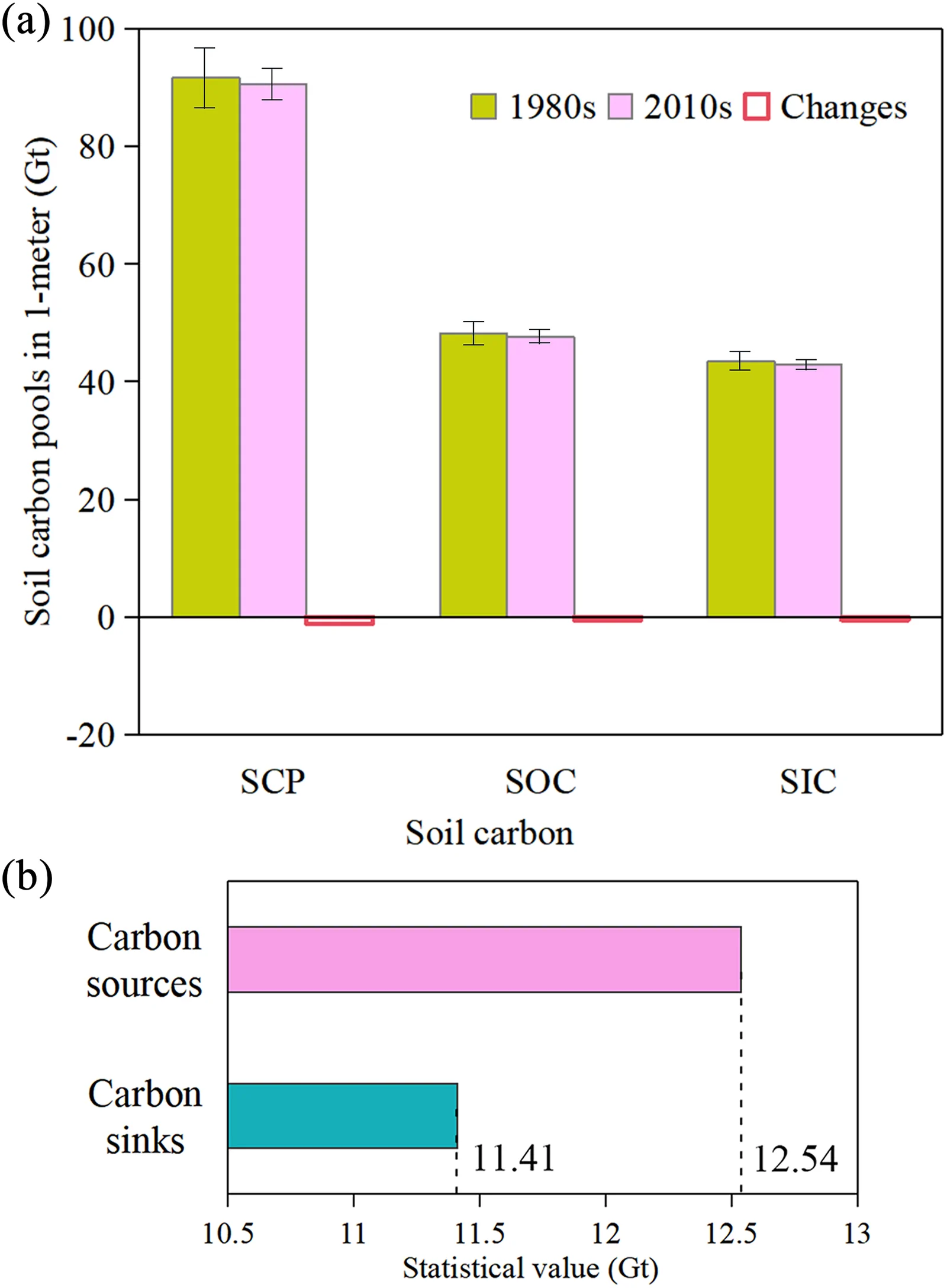
Statistical results for: (a) Soil carbon pools including organic and inorganic carbon and changes in 1 meter soil profile and (b) soil carbon sources and sinks in Northern China.

In Fig 8, the proportion of carbon source areas in Northern China (51.83%) slightly exceeded that of carbon sink areas (48.17%). This macro-level pattern indicates that the overall study area contributes to global net carbon sources. The spatial variation in carbon sources/sinks exhibited a strong coupling relationship with the dominant regional land use types, clearly demonstrating the dominant driving role of land cover in driving carbon dynamics under climate change. In particular, the extensive tracts of UNU in Northwest China with marked increases in SIC, represented significant potential carbon sinks. By contrast, traditional agricultural zones characterized by dense cultivation, such as Northeast China and the Central Plains, experienced widespread concurrent losses of both SOC and SIC, making these regions primary carbon sources. Fig 8 also indicates that some high-altitude cold regions, such as the Qinghai–Tibet Plateau, were significant carbon sources due to the substantial losses of both SOC and SIC.

**Fig 8:**
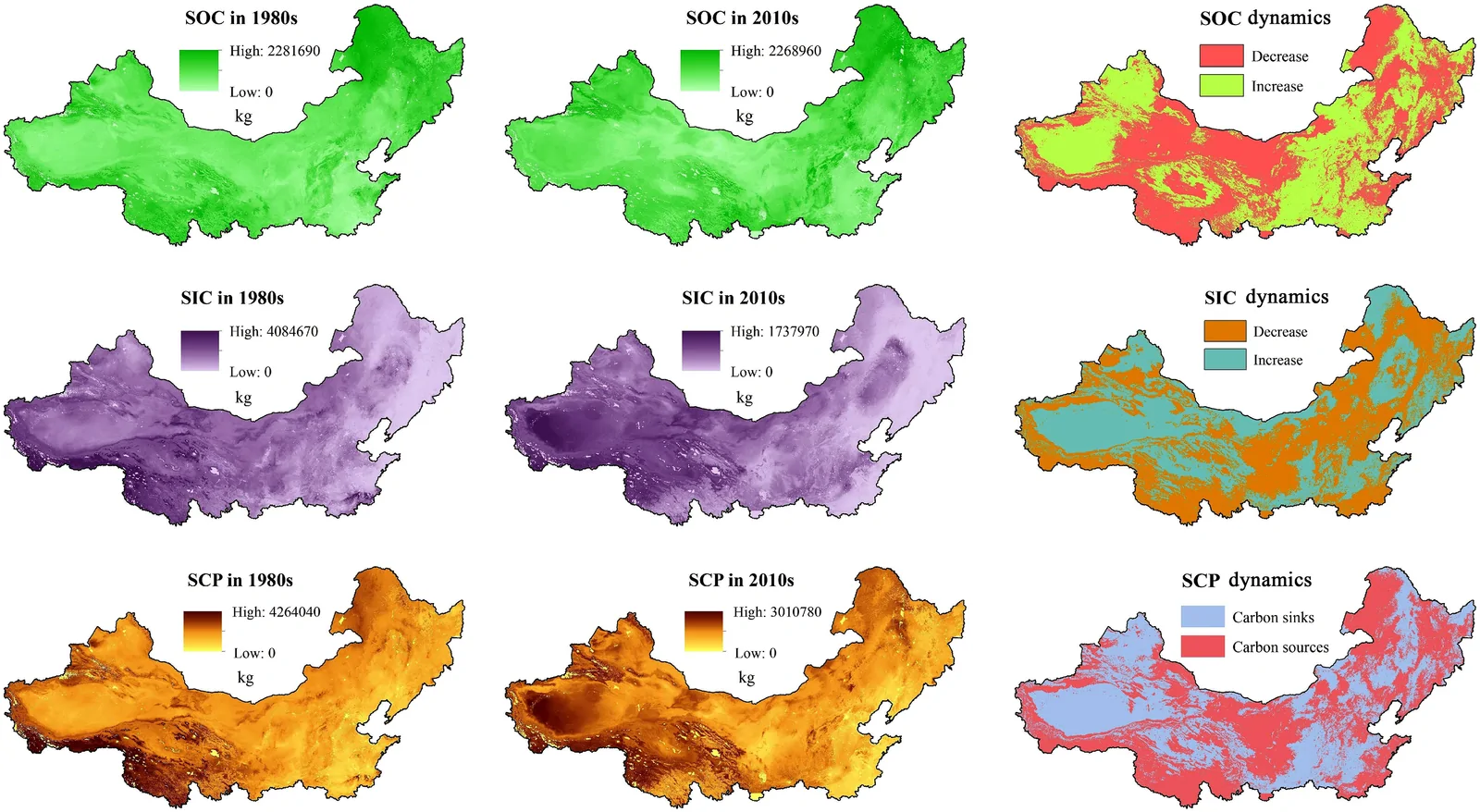
Distribution of soil carbon pools, including organic and inorganic carbon, and changes from the 1980s to 2010s in Northern China.

### SCP dynamics under land use change

#### Soil carbon stock dynamics under land use change.

Despite extensive land use changes in Northern China, the original type was maintained in most of the land areas. Consequently, the carbon dynamics in these land areas continued to represent the primary local soil carbon sources or sinks. Table 4 indicates that GRA constituted a potentially significant carbon source, whereas UNU represented a potential carbon sink due to its pronounced inorganic carbon sink effect. The carbon source and sink effects of CUL and FOR were not significant, where they exhibited SOC sink and SIC source characteristics, respectively.

**Table 4:** Dynamics of SCP under different changes in land use type in Northern China.

Changes in land use type	Changes in SCPs (×10^10^ t)			Carbon pools changes per unit area (t ha ^− 1^)		
	SCP	SOC	SIC	SCP	SOC	SIC
CUL	10.88	52.22	−41.34	1.30	6.23	−4.93
CUL–FOR	0.50	0.70	−0.20	2.13	3.01	−0.87
CUL–GRA	1.13	1.94	−0.81	3.45	5.94	−2.49
CUL–UNU	0.02	0.21	−0.19	0.30	2.98	−2.68
FOR	−11.91	5.31	−17.22	−1.80	0.80	−2.60
FOR–CUL	0.13	0.79	−0.66	0.30	1.80	−1.50
FOR–GRA	4.40	1.70	2.70	8.39	3.24	5.15
FOR–UNU	0.30	−0.47	0.77	1.88	−2.94	4.82
GRA	−165.50	−10.53	−154.98	−11.63	−0.74	−10.89
GRA–CUL	7.76	3.50	4.27	8.66	3.90	4.76
GRA–FOR	1.76	1.42	0.34	2.75	2.22	0.53
GRA–UNU	7.02	−6.14	13.16	3.69	−3.23	6.92
UNU	126.02	−79.78	205.80	8.03	−5.08	13.11
UNU–CUL	5.37	2.75	2.62	16.64	8.51	8.13
UNU–FOR	0.56	0.44	0.12	9.58	7.55	2.03
UNU–GRA	−41.34	−14.05	−27.28	−23.53	−8.00	−15.53

As shown in Table 4, land use changes significantly amplified the magnitude of the total soil carbon dynamics per unit area, making the fluctuations more pronounced, except for GRA. The total carbon dynamics range for undisturbed land ranged from −4.93 to 13.11 t ha^−1^, and those for disturbed land ranged from −23.53 to 16.64 t ha^−1^. Land use change significantly increased the magnitude of the total soil carbon dynamics per unit area by approximately 3.64 t ha^−1^. The SOC pool dynamics in GRA aligned with this trend, but their SIC pools underwent substantial depletion. Even when SOC and SIC accumulation increased through land use conversion, this net carbon deficit remained difficult to offset, indicating that GRA inherently exhibited significant sensitivity and vulnerability, and its role as a substantial carbon sources was potentially linked to climate change.

#### Soil carbon sources and sinks under land use change.

Fig 9 shows that land use change significantly altered the directions of soil carbon sources/sinks in GRA and UNU. UNU was a potential carbon sink, but its conversion to GRA induced significant net carbon losses. By contrast, GRA was a significant carbon source with a decrease of 1.61 Gt but its conversion to UNU resulted in a carbon sink due to significant SIC accumulation. Simultaneously, land use change was a key driver of soil carbon sink formation in CUL and FOR, which was primarily dependent on the SOC pools accumulation of 0.53 and 0.05 Gt. CUL could sequester carbon under management but this often involved SIC losses of 0.41 Gt. Land use conversion could offset or reverse such deficits by enhancing SOC accumulation. FOR soil acted as carbon sources but its carbon sequestration capacity could even significantly improve following conversion, which was largely driven by increases in SOC.

**Fig 9:**
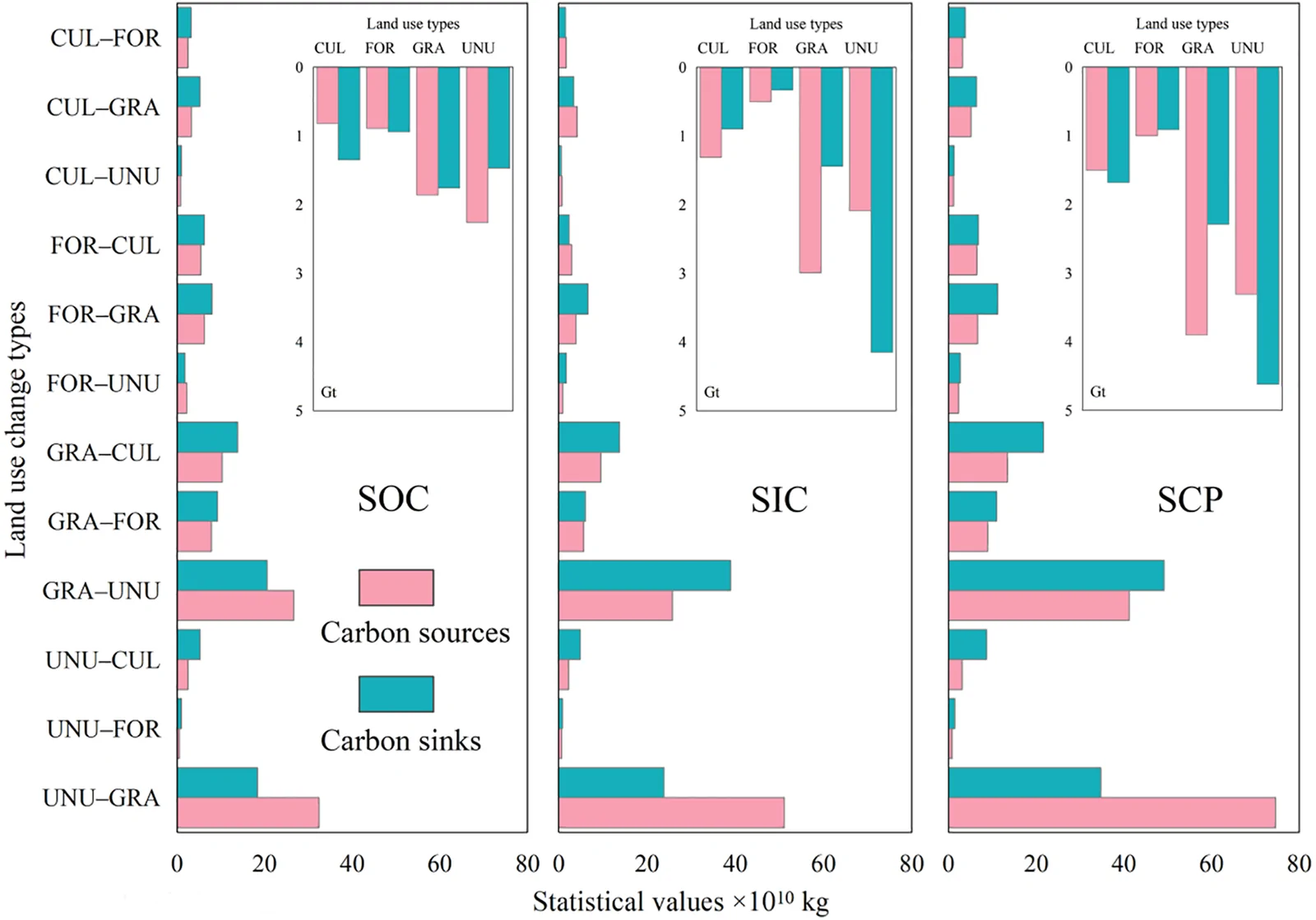
Soil carbon sources and sinks under different land use changes.

### Quantitative association between land use change and SCP

#### Overall direct association between land use change and SCP.

Changes in land use types generally enhanced the responsiveness of SCP to environmental factors, thereby increasing the sensitivity of soil carbon to external environmental stresses. As shown in Figs 6, 10–12, compared with scenarios without land use change, altered land use types result in stronger responses by both SOC and SIC to environmental variables (including land use changes, biology, climate, and nitrogen deposition), with increments in explanatory strength of 0.11 and 0.09, respectively. Under the influence of land use change, the response strength of SOC to pH and patch density increased by 0.2 and 0.21, respectively; and the response strength of SIC to largest patch index, CO_2_ concentration change, and TEM increased by 0.18, 0.15, and 0.14, respectively.

**Fig 10:**
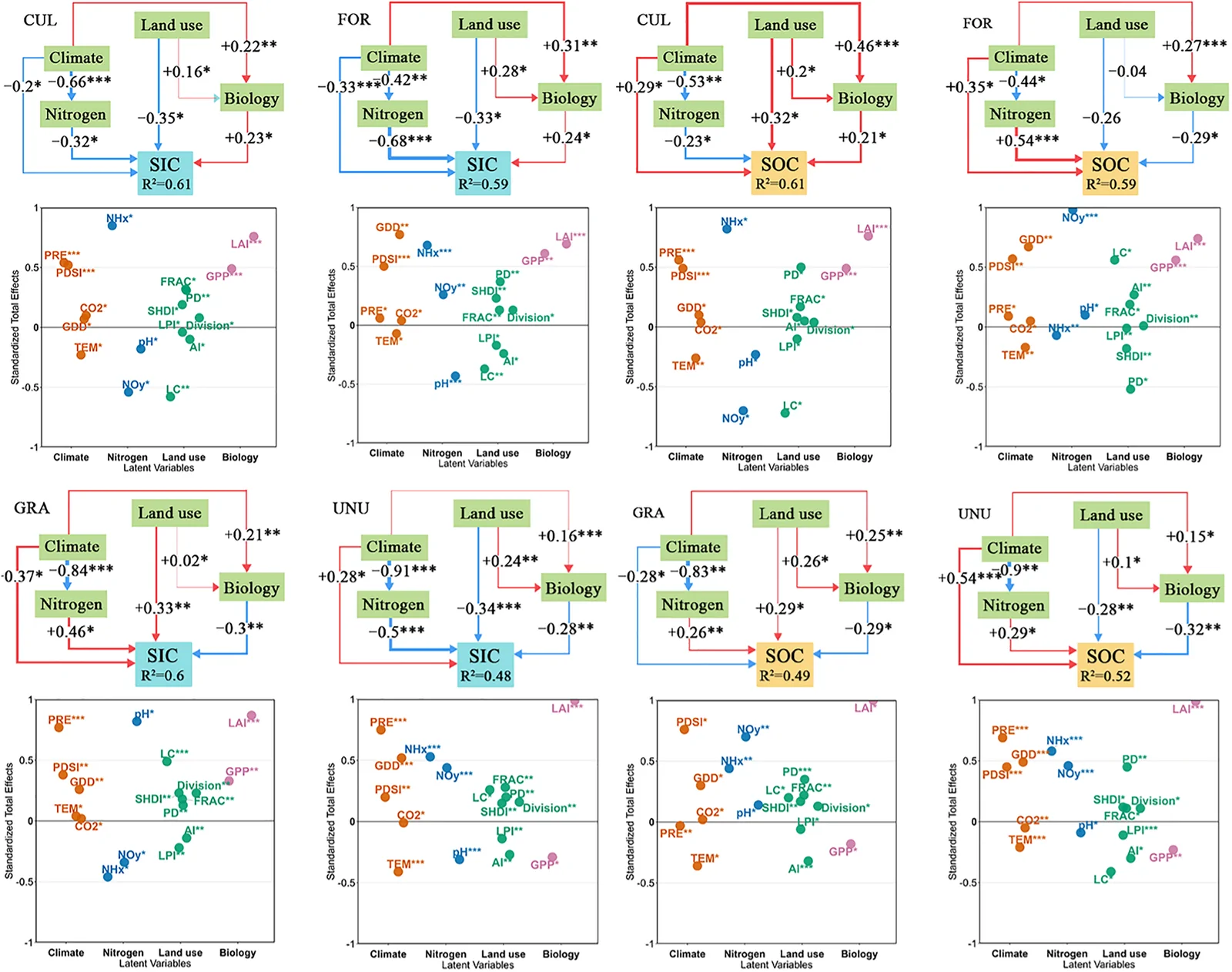
PLS-SEM for soil organic and inorganic carbon under unchanged land conversion scenarios. The observed variables represented by orange dots in the boxes belong to the climate latent variable, blue dots to the soil latent variable, green dots to land use change, and pink dots to the biological latent variable. The dots in the figures below have the same meanings.

**Fig 11:**
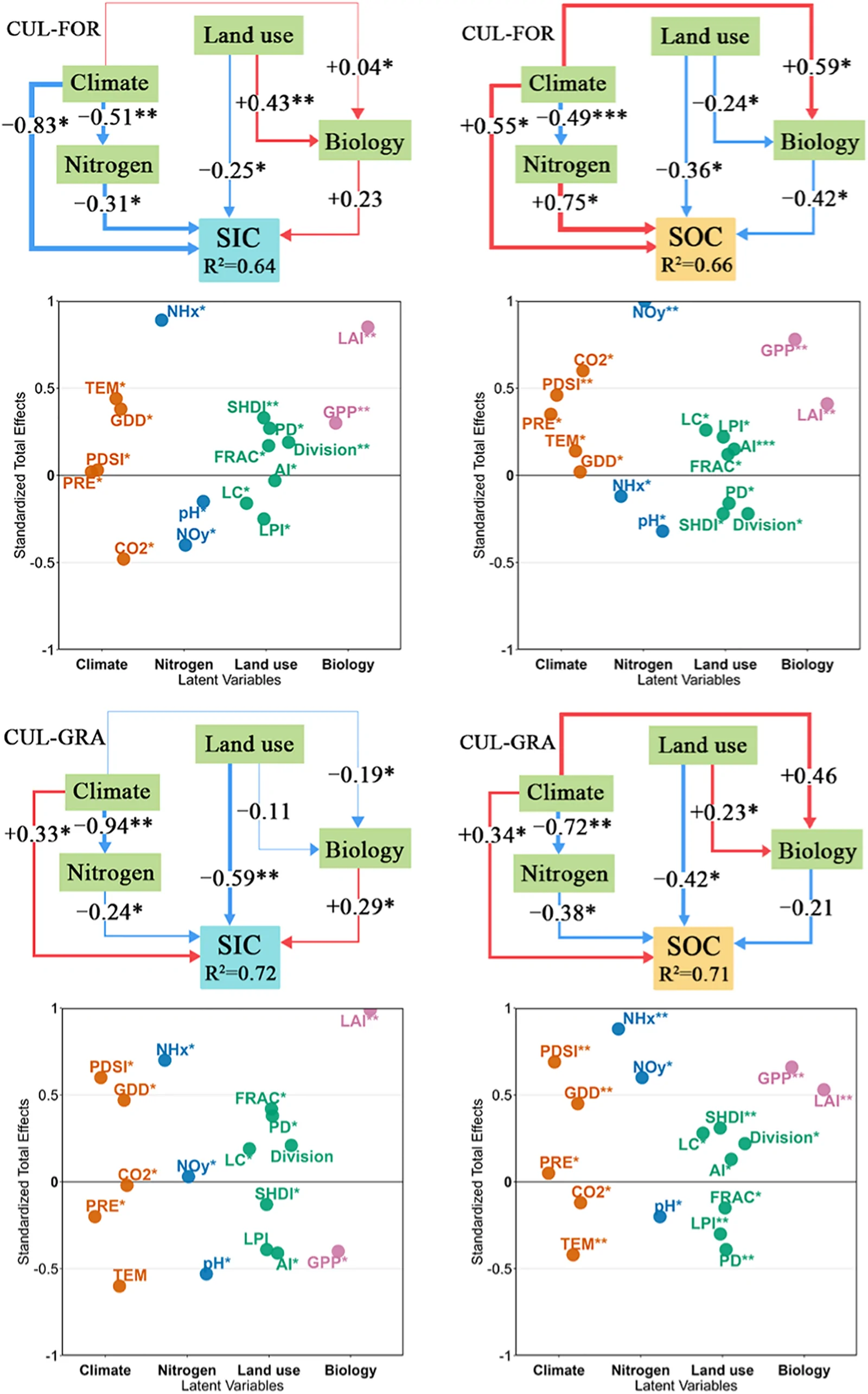
PLS-SEM for soil organic and inorganic carbon under CUL conversion scenarios.

**Fig 12:**
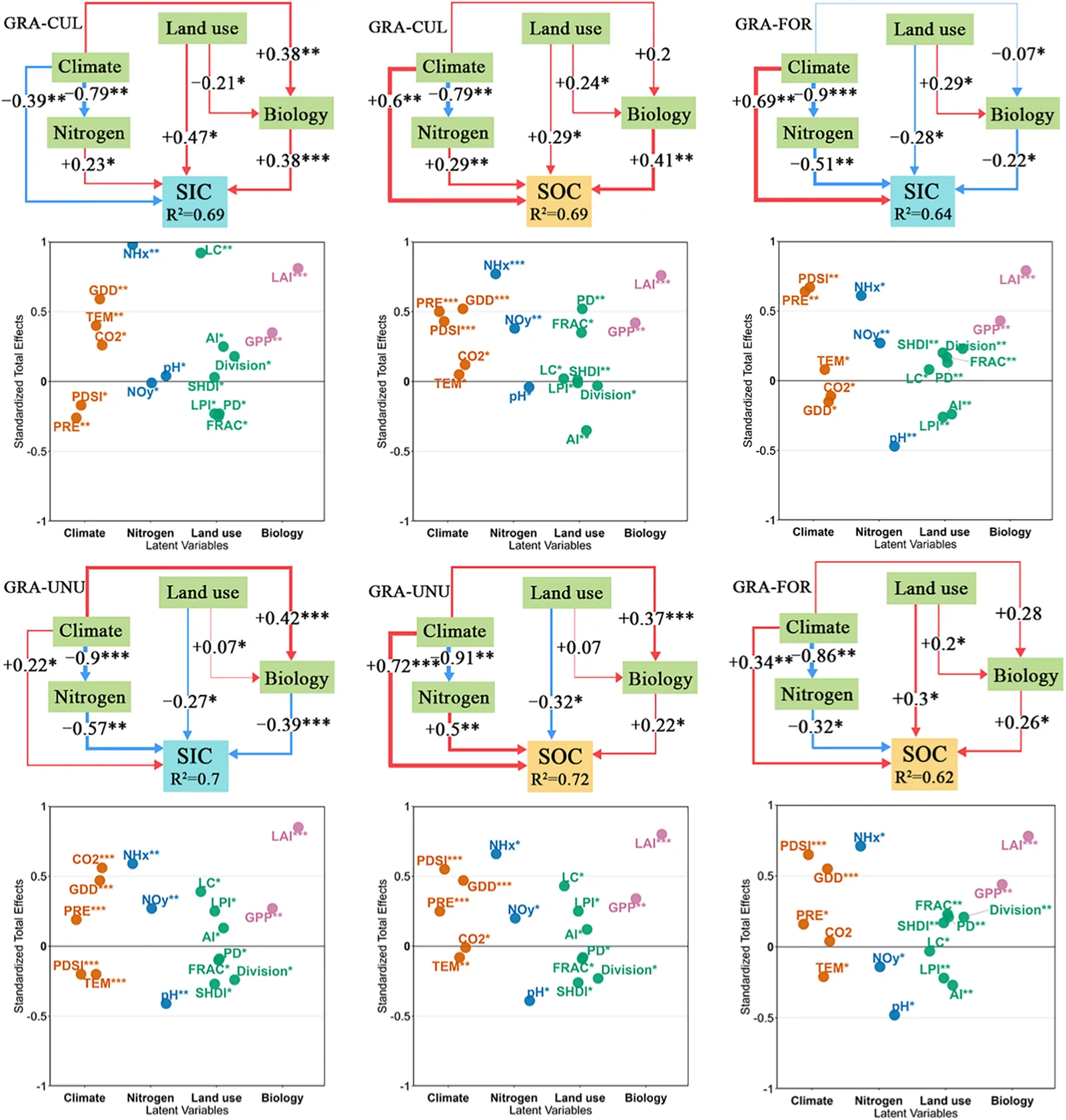
PLS-SEM for soil organic and inorganic carbon under GRA conversion scenarios.

#### Environmental responses of soil carbon under land use change.

Figs 6, 10–12 indicate that land use change not only significantly altered the sensitivity of SCP to environmental changes but also reshaped their specific response patterns. Across different land use types and their conversion processes, both SOC and SIC exhibited systematic changes in the direction and intensity of their responses to environmental factors. Moreover, significant differences were found between various conversion pathways. Models for UNU–FOR, CUL–UNU, and FOR–UNU were excluded due to data sparsity and the consequent inability to develop statistically reliable models. Furthermore, the minor spatial prevalence of these categories suggested their limited overall contribution to the effects under consideration. To substantiate this assessment, we quantified both the SCP dynamics and the areal extents of these three conversion pathways. The total dynamics for UNU–FOR, CUL–UNU, and FOR–UNU account for only 0.79% of the total regional SCP dynamics. Individually, UNU–FOR contributed 0.50%, FOR–UNU contributed 0.27%, and CUL–UNU contributed merely 0.02% of the total soil carbon budget. In terms of spatial extent, these conversions covered 5853.64 km², 7135.48 km², and 15932.93 km², respectively, representing only 0.66%, 0.81%, and 1.80% of the total changed land area, with a combined share of merely 3.27%.

As shown in Fig 11, SOC and SIC exhibited completely different responses to the two changes in CUL. In particular, SOC exhibited a significant positive response to both climate change and nitrogen deposition in scenarios involving afforestation of CUL, whereas SIC exhibited entirely the opposite response. Fig 9 clearly shows that the SCP (particularly the SOC pool) potentially acted as a significant carbon sink in response to increased atmospheric nitrate nitrogen deposition. Furthermore, during the natural succession from abandoned cropland to GRA, SOC and SIC exhibited contrasting responses to this process but both were markedly influenced by latent biological variables, with explanatory strengths of 0.21 and 0.29, respectively. This land use change led to a substantial carbon sequestration in SOC, but a notable loss of SIC. These different responses were intrinsically linked to biological factors, particularly changes in vegetation. SOC responded negatively to biological latent variables, whereas SIC exhibited a significant positive response, implying a critical role for vegetation succession in regulating the dynamic changes in different soil carbon components.

As shown in Figs 6 and 12, the responses of SOC and SIC exhibit significant complexity when FOR undergone alteration. SOC consistently responded positively to nearly all of the environmental variables when FOR was converted to GRA, whereas SIC exhibited a significant negative response. It should be noted that this particular land use change did not alter the fundamental changes in SOC and SIC observed the conversion of FOR to CUL, where SOC constituted a significant carbon source, wheres SIC served as a prominent carbon sink. Our findings further indicated that these divergent changes were closely associated with climatic factors. SOC exhibited a significant positive response to climate change, whereas SIC responded negatively. Critically, the regulation of SCP dynamics by climate change was predominantly governed by the air temperature and aridity.

The conversion processes between GRA and UNU exhibited a pronounced asymmetry in the responses of SOC and SIC, where the SOC content decreased whereas the SIC content increased. The SIC increase was attributed to environmental changes such as reduction of the vegetation leaf area, increased ammonium nitrogen deposition, and elevated CO_2_ concentrations. Conversely, the reduction in SOC was primarily driven by reduced vegetation, increased ammonium nitrogen deposition, and regional drought conditions. By contrast, both SOC and SIC decreased synchronously when UNU was converted back to GRA, where the responses of SOC and SIC to changes in environmental factors were nearly identical in this particular conversion scenario. This synchronized decline was predominantly attributed to a weakened biologically-driven carbon input process combined with the inhibitory effect of increased precipitation on SIC accumulation. Furthermore, the reduction in SIC had a highly significant positive correlation with the pH. Fig 9 indicates that SIC exhibited a significant carbon sequestration effect when both GRA and UNU were converted to CUL. Under these specific conversion scenarios, SOC responded positively to climate change whereas SIC exhibited a negative response, where the dynamics of both carbon pools were strongly regulated by land use transformation. Overall, Figs 6, 10–12 show that land use change profoundly reconfigured the SCP regulatory pathways, thereby significantly altering the sensitivity and specific responses of SOC and SIC to environmental variations.

## Discussion

### Cross-validation of soil carbon in profile and constraints

According to the extrapolation performance analysis in Fig 13, when extrapolating both SOC and SIC from shallow training data (< 40 cm) to a depth of 100 cm, the dispersion between the predicted and observed values gradually increased with the depth, progressively deviating from the 1:1 line, and this was a major limitations of our study. At a soil sampling depth is < 100 cm, the equal-area spline function for SOC exhibited a certain degree of instability between the 1980s and the 2010s, with a tendency to underestimate SOC in the 1980s and to overestimate SOC in the 2010s. By contrast, the equal-area spline function for SIC exhibited stability.

**Fig 13:**
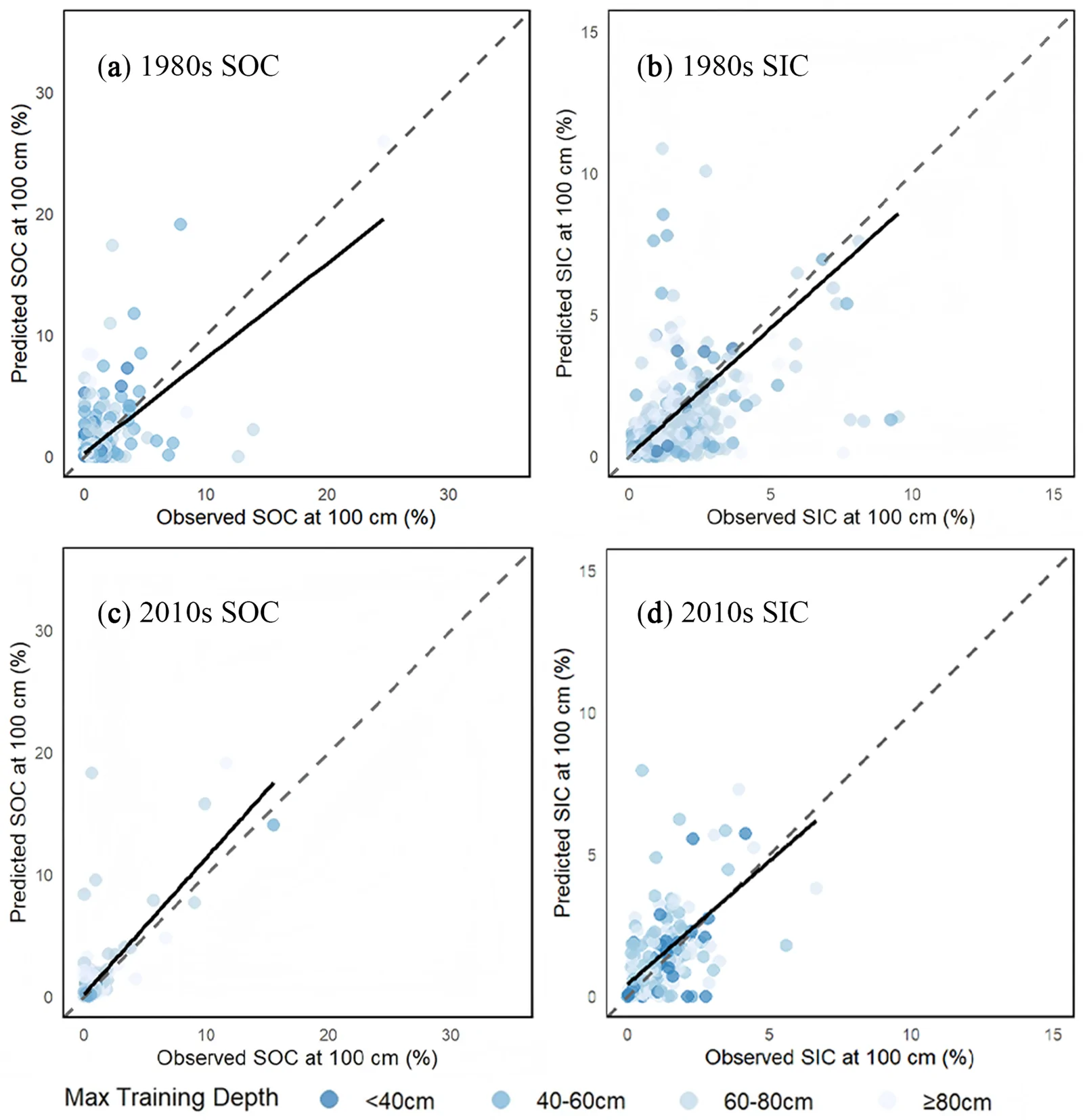
Cross-validation of soil organic and inorganic carbon extrapolation to 100 cm depth.

The equal-area spline function employed in this study does exhibited some fluctuations in concordance (CCC values in Table 5) under certain circumstances, reflecting the complexity of vertical soil carbon distribution and the inherent difficulty of extrapolation but several factors collectively indicate that these limitations did not undermine the overall reliability of the model. These factors include the low systematic bias (Bias in Table 5), the high concordance performance across most depth intervals, and the sufficient and balanced cross-validation samples, as shown by the low percentages in Table 6. Consequently, the model’s predictions remain highly valuable and robust for macro-scale applications such as regional or national soil carbon stock estimation and spatiotemporal trend analysis. Future research may focus on local calibration for specific depth intervals (e.g., 60–80 cm) by introducing stratified covariates or nonlinear basis functions to further improve the point-scale prediction accuracy.

**Table 5:** Numbers of sampling profiles for cross-validation.

Decade	Soil carbon	Index	< 40 cm	40-60 cm	60-80 cm	≥ 80 cm
1980s	SOC	CCC	0.52	0.58	0.37	0.83
		Bias	0.03	0.09	0.11	0.15
	SIC	CCC	0.84	0.32	0.27	0.42
		Bias	–0.13	–0.13	0.10	–0.15
2010s	SOC	CCC	0.45	0.98	0.53	0.88
		Bias	0.05	0.03	0.18	0.14
	SIC	CCC	0.69	0.69	0.38	0.32
		Bias	–0.01	–0.01	0.13	0.11

**Table 6:** Numbers of sampling profiles for cross-validation.

Decade	Soil carbon	Profiles for cross-validation	Sampling profiles	Proportion
1980s	SOC	544	3290	16.53%
	SIC	297	1970	15.08%
2010s	SOC	386	2043	18.89%
	SIC	230	1239	18.56%

Fig 5 showed that the simulated regional means formed narrow distributions and that the separation between the two periods far exceeded the uncertainty within either period. Regional mean SOC and SIC decreased. This indicates that the detected declines in both carbon pools demonstrate a high degree of robustness to depth-extrapolation error.

In addition, the temporal offset between the soil carbon data (1980s and 2010s) and the land use change snapshots (1985 and 2015) introduces uncertainty regarding the time-lag effect of soil carbon response; while our 30-year comparison window captures the dominant directional trends, the magnitude of carbon stock change may be conservative as soil carbon pools may not have fully equilibrated with the converted land use types. Furthermore, both the 1980s and 2010s SOC datasets were determined using the same Van Bemmelen factor. Consequently, methodological error enters both period estimates as an identical multiplicative term that cancels when temporal differences and relative source/sink transitions are computed. Residual recovery variation behaves as random spatial noise rather than temporal systematic bias, and is bounded by our validation errors in Tables 7 and 8. Nevertheless, Walkley-Black estimates method may underestimate absolute SOC stocks, and our conclusions therefore concern the direction and relative magnitude of change rather than absolute pool sizes. Finally, our analysis is restricted to natural and semi-natural land covers. Urban and built-up soils were excluded because construction land is severely underrepresented in the national soil profile archive, and its sealed, anthropogenically mixed substrates require dedicated sampling designs. Urban soils represent a potentially substantial but unquantified component of the regional budget and should be a potential for dedicated future investigation.

**Table 7:** R^2^, RSME, and MAE values for RF prediction results after important factor selection.

Soil carbon	Decade	Set	R^2^	Average content (g kg^–1^)	RSME (g kg^–1^)	MAE (g kg^–1^)
SOC	1980s	Training set	0.81	4.54	0.48	0.26
		Prediction set	0.46		0.56	0.35
	2010s	Training set	0.87	4.47	0.43	0.25
		Prediction set	0.57		0.48	0.32
SIC	1980s	Training set	0.87	3.95	0.49	0.25
		Prediction set	0.51		0.65	0.38
	2010s	Training set	0.89	3.88	0.38	0.19
		Prediction set	0.62		0.40	0.22

**Table 8:** Spatial cross-validation results.

Metrics	Decade			
	1980s		2010s	
	SOC	SIC	SOC	SIC
RMSE (%)	3.12	2.51	2.95	2.87
MAE (%)	1.11	1.84	1.15	1.86
R²	0.36	0.35	0.41	0.34

### Evaluation and spatial cross-validation of RF model

After feature selection, the predictive accuracy of the RF model was high for both SOC and SIC (Table 7). The performance with the training set was robust (R² = 0.81–0.89), and that with the prediction set maintained low absolute errors (RMSE = 0.40–0.65 g kg^−1^; MAE = 0.22–0.38 g kg^−1^), corresponding to relative errors of approximately 10–16%. Although prediction R² declined to 0.46–0.62, this was primarily attributable to the narrower variance range in the independent validation set rather than model degradation. Critically, the RMSE values for predictions increased by only 5%–33% compared with the training set, indicating strong generalization capability without error propagation.

Based on the SOC and SIC sampling data and the top 10 most important environmental covariates for the 1980s and 2010s, the optimal numbers of clusters were determined as three and four, respectively. Accordingly, Northern China was divided into three and four spatial blocks for the 1980s and 2010s, respectively, to conduct spatial block cross-validation as shown in Fig 14. To facilitate comparison across soil carbon types and survey periods with differing mean concentrations, spatial cross-validation metrics are expressed as relative errors. The results indicated stable model performance across all spatial blocks, with relative RMSE ranging from 2.51% to 3.12%, relative MAE from 1.11% to 1.86%, and R² from 0.34 to 0.41 (Table 8). These results indicate that under rigorous validation conditions accounting for spatial heterogeneity and autocorrelation, the model achieved moderate spatial generalization. While this level of performance supports macro-scale applications such as regional soil carbon stock estimation and long-term trend analysis across Northern China, the relatively modest explanatory power in spatial extrapolation suggests that point-scale predictions should be interpreted with caution, particularly in data-scarce regions.

**Fig 14:**
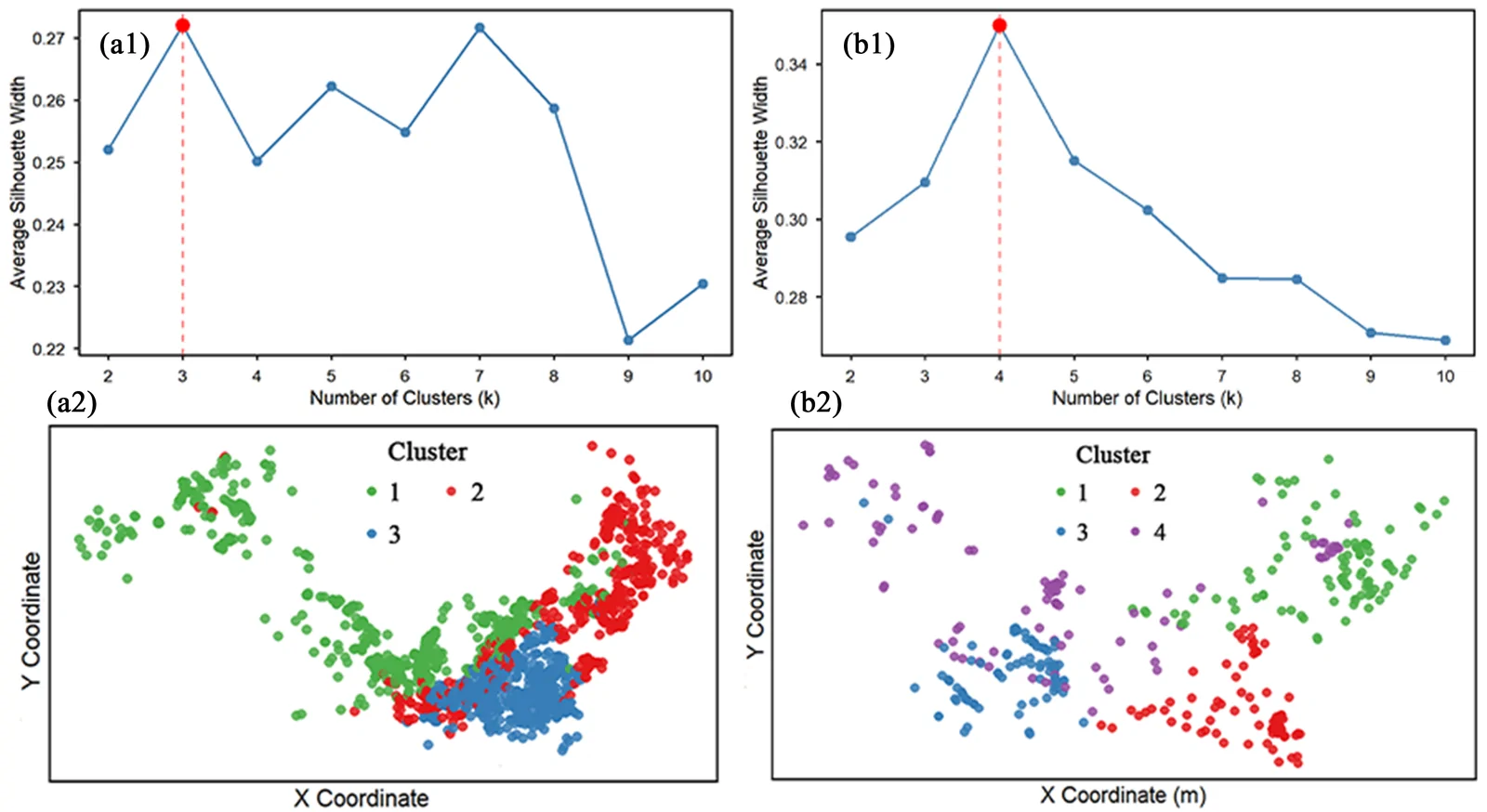
Spatial cross-validation results: (a1) and (b1) are contour coefficient diagrams, (a2) and (b2) are clusters of sample points. (a) the 1980s and (b) the 2010s.

### Evaluation of collinearity and effect of PLS-SEM

The PLS-SEM employed in this study is a PLS form of SEM, where the fundamental framework is SEM. In SEM, the path coefficients describing latent variable relationships are obtained by estimating a series of regression equations. Therefore, it is necessary to examine the multicollinearity among explanatory variables to avoid its impacts on subsequent path coefficient analysis [47]. VIF is a commonly used metric for assessing multicollinearity. Strictly speaking, a VIF value exceeding 5 indicates a significant risk of multicollinearity, and values between 3 and 5 may also suggest its presence. Values below 3 denote an acceptable model. As shown in Fig 15, the average VIF was 2.59 (VIF < 3) for the PLS-SEM model constructed in this study, indicating no significant multicollinearity among the variables employed, thereby ensuring the stability and reliability of the path coefficient estimates.

**Fig 15:**
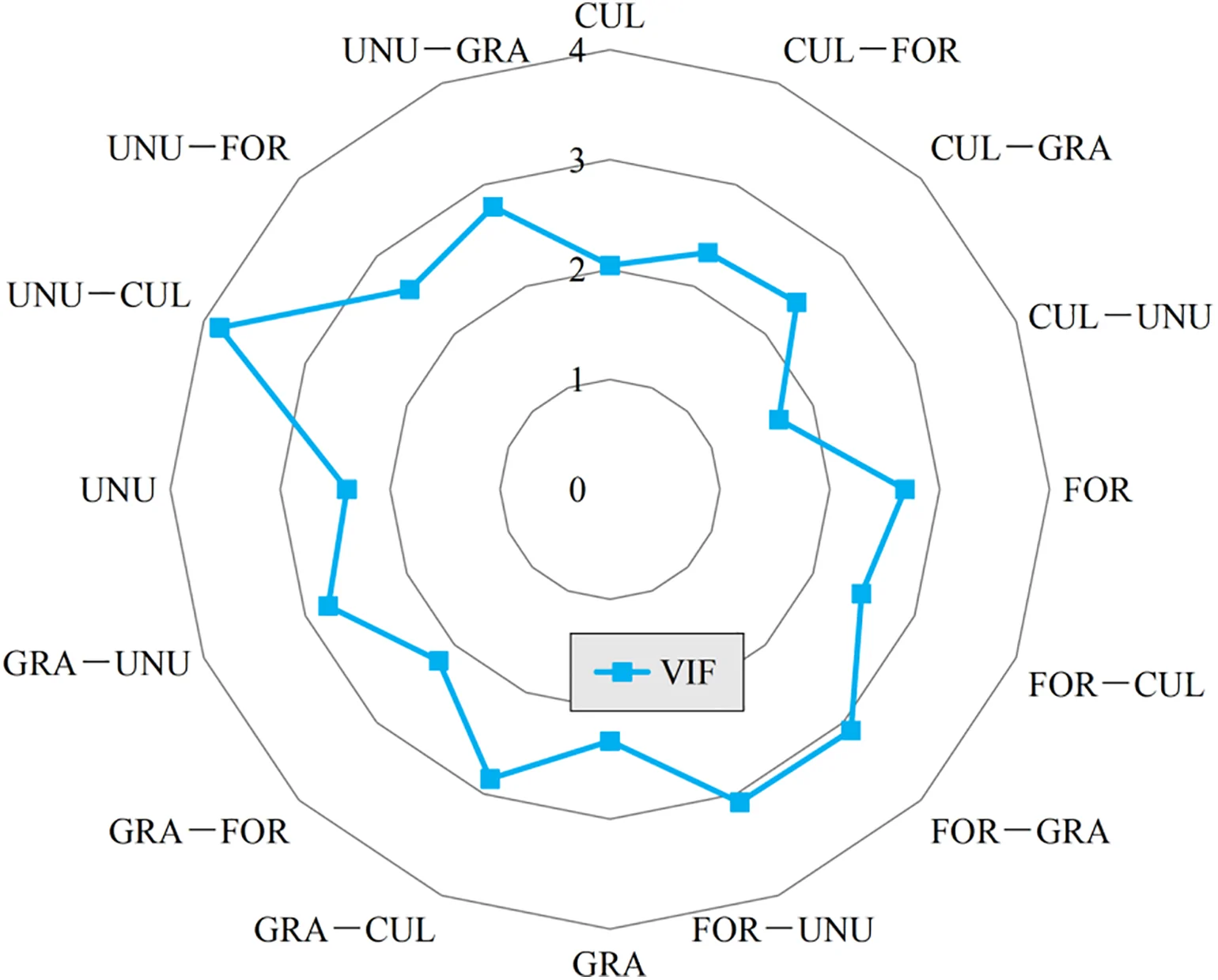
VIF values for PLS-SEM under different land use change types.

As shown in Table 9, internal consistency of the PLS-SEM measurement models was satisfactory, with Cronbach’s α ranging from 0.69 to 0.88 (mean = 0.77) for SOC and 0.66 to 0.87 (mean = 0.76) for SIC, and composite reliability exceeding 0.70 in the majority of cases. Convergent validity was supported by AVE values above 0.50 for most scenarios (mean = 0.55 for both carbon pools). Discriminant validity (HTMT < 0.85) and model fit (SRMR < 0.08) were generally acceptable, particularly for high-explanatory-power scenarios (R² ≥ 0.70), all of which passed every evaluation criterion. While a few low-R² scenarios exhibited marginal measurement quality, the overall results confirm that the construct operationalization is robust for the dominant land use transitions.

**Table 9:** Model evaluation of PLS-SEM.

Land use change	SOC					SIC				
	α ^a^	CR ^a^	AVE ^a^	HTMT ^a^	SRMR ^a^	α ^a^	CR ^a^	AVE ^a^	HTMT ^a^	SRMR ^a^
CUL	0.78	0.85	0.57	0.82	0.07	0.76	0.78	0.58	0.82	0.08
CUL–FOR	0.75	0.85	0.58	0.79	0.07	0.76	0.8	0.56	0.81	0.07
CUL–GRA	0.86	0.88	0.61	0.76	0.07	0.87	0.89	0.63	0.78	0.06
CUL–UNU	0.70	0.72	0.52	0.88	0.09	0.71	0.77	0.52	0.83	0.08
FOR	0.76	0.78	0.53	0.84	0.08	0.7	0.76	0.51	0.88	0.09
FOR–CUL	0.74	0.76	0.49	0.84	0.08	0.72	0.78	0.51	0.84	0.08
FOR–GRA	0.83	0.90	0.60	0.82	0.07	0.83	0.85	0.65	0.79	0.07
FOR–UNU	0.69	0.73	0.48	0.88	0.08	0.71	0.76	0.51	0.85	0.08
GRA	0.69	0.71	0.46	0.91	0.09	0.79	0.83	0.55	0.81	0.07
GRA–CUL	0.75	0.79	0.52	0.82	0.07	0.79	0.84	0.54	0.81	0.08
GRA–FOR	0.77	0.79	0.54	0.79	0.08	0.81	0.83	0.58	0.83	0.07
GRA–UNU	0.87	0.89	0.65	0.81	0.06	0.83	0.86	0.62	0.80	0.07
UNU	0.74	0.76	0.51	0.83	0.08	0.7	0.72	0.48	0.91	0.09
UNU–CUL	0.88	0.91	0.61	0.77	0.06	0.84	0.86	0.63	0.77	0.06
UNU–FOR	0.77	0.84	0.54	0.79	0.07	0.70	0.75	0.51	0.85	0.08
UNU–GRA	0.74	0.77	0.52	0.83	0.08	0.66	0.68	0.45	0.91	0.09

^a^The evaluation indicators reported in the table are the means of the evaluation indicators corresponding to each latent variable within the measurement model. Since the complete set of results is too extensive, only the means are presented here as a concise summary.

R² and Q² reflect the predictive effect size and predictive correlation of PLS-SEM, respectively. Based on the results in Table 10, the R² values for endogenous latent variables in this study ranged from 0.27 to 0.83. The mean R² values for SOC and SIC were 0.46 and 0.43, respectively, and thus both exceeded the critical threshold of 0.1, indicating that the PLS-SEM model had strong explanatory power and could effectively capture the response of SCPs to land use and environmental changes. In addition to analyzing the uncertainty coefficients for endogenous latent variables, Q² was employed to evaluate the predictive performance of the research model [47]. In general, a Q² value greater than zero indicates predictive relevance whereas a value less than zero suggests that the model lacks predictive relevance. In this study, all of the Q² values exceeded zero, with mean values of 0.35 and 0.37 for SOC and SIC, respectively, demonstrating that the PLS-SEM model employed in this study had acceptable predictive relevance.

**Table 10:** The R² and Q² values for PLS-SEM in different land use types.

Changes in land use type	SOC		SIC	
	R² ^a^	Q²	R² ^a^	Q²
CUL	0.29	0.27	0.27	0.25
CUL–FOR	0.39	0.29	0.42	0.45
CUL–GRA	0.52	0.39	0.48	0.32
CUL–UNU	0.83	0.61	0.73	0.62
FOR	0.32	0.43	0.30	0.39
FOR–CUL	0.39	0.59	0.43	0.33
FOR–GRA	0.44	0.24	0.44	0.24
FOR–UNU	0.75	0.72	0.60	0.77
GRA	0.29	0.22	0.29	0.23
GRA–CUL	0.39	0.21	0.31	0.34
GRA–FOR	0.40	0.22	0.40	0.25
GRA–UNU	0.42	0.26	0.38	0.23
UNU	0.33	0.27	0.33	0.23
UNU–CUL	0.51	0.28	0.48	0.41
UNU–FOR	0.72	0.52	0.74	0.70
UNU–GRA	0.36	0.22	0.34	0.22
Average	0.46	0.35	0.43	0.37

^a^Models with R² < 0.40 but R² > 0.10 fall within the weak explanatory range, and their path coefficients should be interpreted cautiously.

It is important to emphasize that the R² values for single land use type contrast sharply with the substantially higher values observed for conversion pathways, particularly those involving unused land. This divergence reflects a scale-dependency in driver dominance within a single land use type, soil carbon is strongly modulated by fine-scale management factors (irrigation, fertilization, grazing intensity, tillage) that are highly heterogeneous and largely unobserved at the regional extent, leaving substantial residual variance [50]. Previous studies at the county scale have demonstrated that population density and local agricultural management can explain over 30% of SOC variance, often exceeding the explanatory power of climate and topography [51]. However, such management variables are not available as spatially continuous, long-term datasets for Northern China, and their omission accounts for the unexplained variance in CUL and GRA models. By contrast, land use conversion constitutes a strong directional perturbation that reorganizes carbon pools along macro-scale biophysical gradients, making the response more predictable from the climate, terrain, and soil variables captured in our models. Thus, the lower R² for CUL and GRA does not indicate model deficiency, but rather the inherent limitation of explaining management-driven micro-heterogeneity with coarse-resolution regional predictors [52].

### A significant net soil carbon source in Northern China

The interpretation of our findings requires careful consideration of the 30-year temporal scale of arid-region soil carbon dynamics. The national soil survey data employed in this study provide bulk SOC stocks without physical or chemical fractionation into labile, slow, or mineral-associated pools. In the semi-arid and arid soils, this bulk measurement is dominated by relatively stable, mineral-associated organic carbon, which is inherently buffered against rapid change by strong organo-mineral interactions and long-term pedogenic controls. From this perspective, the statistically significant changes in SOC stock distribution and vertical allocation observed across the two survey periods are particularly noteworthy. They indicated that land use change has already perturbed the soil carbon system sufficiently to overcome the high background spatial variance imposed by parent material heterogeneity. Furthermore, the concurrent changes in SIC stocks, which respond more sensitively to decadal-scale perturbations in hydrological regimes, irrigation, and CO_2_ dynamics, provide complementary evidence that land use change has initiated measurable carbon cycle reorganization within this timeframe. We therefore interpret our results as capturing a transient, management-sensitive phase of soil carbon reconfiguration rather than a pedogenic equilibrium. These early-stage trajectories are critical for land management decision-making, as they reveal the directional sensitivity of SCPs to land use change before long-term stabilization processes fully manifest.

Northern China was a stable net soil carbon source during the study period, with the same decreases in both SOC and SIC, indicating that soil carbon losses did not occur only due to changes in a single carbon pool but instead from the combined effects of coordinated responses across SCP. Consequently, Northern China remains a net contributor to terrestrial carbon emissions under current global climate and land use changes, imposing direct constraints on regional carbon neutrality pathway assessments. This coordinated depletion of both organic and inorganic pools carries critical implications for regional carbon management: it suggests that carbon mitigation strategies focusing exclusively on SOC enhancement (e.g., through soil amendments or land retirement) may be insufficient, as concurrent SIC losses could offset overall soil carbon gains. Therefore, effective carbon neutrality pathways in Northern China must adopt a dual-pool accounting framework rather than relying on single-pool assessments. This result is consistent with our assumption (1).

Existing research predominantly highlights increasing trends in SOC within surface soils of northern hemisphere farmlands [53,54], and the findings of increased SOC in cropland from this study are consistent with those existing results. Some studies have also demonstrated profile differentiation in SOC dynamics. For instance, SOC increased in the 0–20 cm layer of paddy soils on the Chengdu Plain but decreased in deeper layers from the 1980s to the 2010s [55], and also increased in the 0–40 cm layer on the Guanzhong Plain but decreased in the 40–100 cm layer [29]. These study areas contained extensive intensively CUL under active human regulation and focus on SOC variations at different soil depths, but they are inapplicable to large-scale regions dominated by natural and semi-natural systems. Northern China is characterized by GRA and UNU as the primary land use types, and distinct patterns were found when analyzed at the 1-m soil profile scale. GRA was a strong net carbon source, whereas UNU represented a substantial carbon sinks, highlighting the dominant regulatory role of land use types in shaping soil carbon sources/sinks patterns. This conclusion shows the need to clearly distinguish between different land use types and their corresponding soil carbon responses and mechanisms when assessing terrestrial ecosystem carbon sources and sinks.

### Land use change enhanced soil carbon responses

Previous studies have established that land use change significantly alters soil carbon dynamics, but most have focused on single carbon pools or specific conversion types without disentangling the divergent responses of SOC and SIC to environmental drivers [10,56–58]. This study reveals that irrespective of the specific conversion pathway, transitions from CUL consistently result in SOC accumulation alongside SIC reduction. Specifically, during these CUL outflow processes, the positive response of SOC to climate change is substantially enhanced, primarily driven by increased precipitation and a shift towards wetter climatic conditions. This finding is consistent with prior work demonstrating that moisture enrichment benefits SOC sequestration but inhibits SIC stabilization [59]. However, we extend these earlier findings by showing that long-term intense tillage disturbances characteristic of historical CUL management, coupled with soil acidification induced by atmospheric nitrogen deposition, likely exacerbate carbonate dissolution and leaching [35,60]. Thus, by integrating land use history with biogeochemical processes, our results provide a mechanistic explanation for the observed phenomenon that SOC accumulation in CUL systems is systematically accompanied by SIC loss.

Regarding FOR–GRA conversion, existing research suggests that highly productive natural vegetation types favor synergistic carbon sequestration across multiple pools [61,62]. Our findings corroborate and extend this view that such conversion enhances accumulation in both carbon pools, primarily driven by sustained carbon inputs from increased leaf area index and gross primary productivity alongside relatively stable soil chemistry. In contrast to prior studies that have emphasized biotic controls on SOC, our results further reveal that after FOR transitions to CUL or UNU, SOC remains primarily positively driven by biotic factors, whereas SIC exhibits heightened sensitivity to climate and shows a declining trend. This distinction underscores a critical insight from our work, namely that human disturbance poses distinct risks to SIC stability that have been insufficiently addressed in previous research.

Extensive GRA degradation in Northern China has been caused by overgrazing [63], with declining vegetation productivity and soil erosion leading to substantial soil carbon loss. Our results demonstrate that soil carbon sources in northern GRA were characterized by the combined loss of SOC and SIC, where the loss of SIC was dominant. Path analysis based on PLS-SEM indicated that climate change was a key driver of inorganic carbon loss from GRA soils, and this climatic signal was primarily dominated by reduced precipitation and a decreased PDSI. Decreased precipitation directly weakens GRA water regulation and carbon sequestration functions [64]. Even under high-emission scenarios, the enhanced water use efficiency resulting from elevated atmospheric CO_2_ concentrations can hardly offset the severe negative impacts of drought on SCP, demonstrating that the inherent vulnerability of GRA, combined with external climatic stresses, collectively leads to the loss of SCP dominated by SIC.

The results show that UNU as a vast potential carbon sink in Northern China, but it also reveals a concurrent increase in both SOC and SIC after UNU–CUL conversion. This result is a pattern that contradicts not only our speculation (2) but also the prevailing view in the literature that cropland expansion depletes SIC stocks through tillage-induced disturbance and enhanced leaching [65,66]. The PLS-SEM analysis offers a mechanistic basis for this divergence. For SIC, the path from UNU–CUL to biological variables indicated that the establishment of CUL vegetation stimulated biological activity, which in turn promoted SIC accumulation. This aligns with the biological carbonate pump operating in arid ecosystems that enhanced root and microbial respiration elevates soil CO_2_ concentrations, facilitating carbonate dissolution in the rhizosphere, subsequent capillary rise and evaporative concentration then precipitate secondary carbonates in surface horizons [67,68]. N-deposition were strongly positively linked to SIC suggests that irrigation with Ca² ⁺ /HCO_3_^−^-rich water and nitrogen fertilization in newly converted CUL likely shifted soil chemical equilibria toward carbonate precipitation, overriding the SIC-depleting effects of tillage documented in other agroecosystems [69]. Notably, this mechanism applies specifically to the converted CUL, whereas the SIC accrual observed in UNU itself cannot draw on irrigation or fertilization and must therefore be sustained by aeolian Ca supply. At the observed accrual rate of 2.68 g C m^-2^ yr^-2^ (8.03 t ha^-1^ over 30 years), the stoichiometric Ca demand for ~22 g CaCO_3_ m^-2^ yr^-1^ is consistent with measured dust-borne carbonate deposition in this region (11–54 g CaCO_3_ m^-2^ yr^-1^) [70,71]. For SOC, the observed increase was primarily driven by anthropogenic carbon inputs [72,73]. In contrast to studies that identify SIC as a sensitive early indicator of land use change, our findings suggest that in severely degraded arid lands, anthropogenic inputs (irrigation and fertilization) and biological carbonate pumping can transiently mask the SIC-depletion signal, rendering SIC a poor early-warning indicator in such contexts.

In general, our findings reveal that soil carbon responses to global change were shaped by land use type and require explicit consideration of both organic and inorganic pools. By demonstrating divergent SOC and SIC dynamics across natural, semi-natural, and managed systems, this study challenges the conventional focus on surface SOC in farmlands and underscores the critical role of SIC in regional carbon budgets, particularly in arid and semi-arid zones. Practically, the stark contrasts between carbon sources and sinks highlight that carbon neutrality strategies must be land use-specific and should safeguard GRA from degradation and drought, harness UNU sequestration potential, and acknowledge SIC losses accompanying agricultural transitions. Incorporating such dual-pool, land use-differentiated frameworks into carbon accounting and climate mitigation planning is therefore essential for realistic regional carbon neutrality assessments.

### Land use change reversed soil carbon responses

Results of this study demonstrate that land use change can reshape soil carbon dynamics as our speculation (3), primarily manifested in two main ways. First, altering land use types can reverse regional carbon losses due to the strong coupling between soil carbon sources-sinks and regional land use type changes. This transformation can shift the carbon source patterns for GRA, endowing them with significant carbon sink potential. After land use conversion from GRA (to CUL, FOR, or UNU), soil carbon sinks may form through synergistic changes in the SOC and SIC pools. In particular, this transformation frequently reverses the response of SIC to precipitation from positive to negative, thereby exacerbating its sensitivity. This reversal likely reflects a shift in the dominant hydrological control on carbonate dynamics. Moderate precipitation may favor carbonate precipitation via reduced leaching and evaporative concentration under GRA. However, altered soil hydrology and root water uptake may lower soil water potentials below thresholds required for Ca²⁺ and HCO_3_^−^ transport after GRA conversion, particularly to CUL or FOR, disrupting precipitation kinetics and rendering SIC more vulnerable to drought-induced depletion [10,74,75].

Moreover, it promotes the shift of SOC toward positive feedback in response to improved biotic conditions. These two processes collectively mitigate the loss of the regional SCP loss. In general, increased CO_2_ concentrations enhance the partial pressure, promoting soil carbonate formation, thereby increasing the accumulation of inorganic carbon sources [66]. However, during the conversion from GRA to CUL or FOR, increases in vegetation cover and photosynthetic carbon uptake often occur simultaneously, enhancing the biological utilization of atmospheric CO_2_. Thus, the soil carbon sink associated with GRA conversion was primarily linked to the combined associations of climate change and improved biotic conditions, which is consistent with the pattern shown in Fig 13. In the future, optimizing the functional composition of grassland plants and enhancing the structural and functional resilience of communities will help strengthen the stability of GRA carbon pools and mitigate the risk of carbon loss under climate change. This approach will guide GRA ecosystems toward enhanced carbon sink functions under appropriate human intervention.

Second, our results demonstrate that land use change not only reversed soil carbon sources and sinks but also played a crucial role in amplifying soil carbon dynamics per unit area. After land use conversion, the magnitude of the total soil carbon change increased significantly. In addition, both SOC and SIC exhibited stronger responses to environmental variables, including land use, biological factors, climate, and nitrogen deposition. Thus, land use change altered the direction of soil carbon sources/sinks but also heightened the variability and uncertainty of SCP. Land use conversion markedly increased the sensitivity of soil carbon dynamics to climate change, where SOC primarily responded to climate shifts whereas SIC was more strongly driven by the CO_2_ concentration. The response of SOC to climate change markedly intensified after land use conversion, reflecting the high sensitivity of the vegetation cover, litter input, and microbial processes to temperature and moisture variations [67,68,72]. By contrast, SIC exhibited a significantly heightened response to changes in the atmospheric CO_2_ concentration, with a particularly marked increase in the sensitivity of its leaching-precipitation balance in response to variations in atmospheric CO_2_ partial pressure. Land use changes further strengthened the coupling between SIC and atmospheric carbon by influencing the soil carbonate leaching-precipitation balance and soil hydrological processes. This functional division of labor between SOC and SIC in terms of their primary controlling factors was difficult to discern in previous studies that often analyzed them together.

## Conclusions

This study reveals that Northern China functioned as a net soil carbon source from the 1980s to the 2010s, driven by coordinated SOC and SIC losses. Land use type was strongly associated with carbon source/sink patterns, with GRA acting as a source via SIC depletion and UNU as a sink through SIC accumulation. Notably, SOC responded primarily to biotic drivers, whereas SIC showed heightened sensitivity to climate and CO_2_ fluctuations, revealing a functional division between pools. These findings challenge the prevailing single-pool paradigm and underscore the necessity of dual-pool accounting in terrestrial carbon assessments. From a management perspective, differentiated strategies are needed: controlling grazing to mitigate SIC loss in GRA and prioritizing ecological restoration to harness SIC sequestration in UNU. Regional carbon neutrality frameworks must explicitly incorporate SIC to avoid underestimating land use effects. We acknowledge greater uncertainty in deeper layer predictions below 80 cm and limited inter-annual resolution. Future work should employ stratified covariates and process-based models to improve accuracy and validate these pathways at broader scales.
